# Supplementary material for: Association between vitamin D receptor gene polymorphism and essential hypertension: An updated systematic review, meta-analysis, and meta-regression
Source: PLoS One. 2024 Dec 23;19(12):e0314886. doi: 10.1371/journal.pone.0314886 (PMC11666036; doi:10.1371/journal.pone.0314886)
Supplement: S1 Table — (DOCX) [file pone.0314886.s001.docx]

| **Database** | **Keyword** | **Number of Studies** |
| --- | --- | --- |
| PubMed | (((Hypertension[MeSH Terms]) OR ("Arterial Hypertension" OR "Essential Hypertension")) AND ((Vitamin D[MeSH Terms]) OR (Cholecalciferol OR Ergocalciferol))) AND ((((Gene Polymorphism[MeSH Terms]) OR (Single Nucleotide Polymorphism[MeSH Terms]))) OR (("Gene Variation" OR "Genetic Variation" OR "Polymorphism" OR SNP) OR ("rs2228570" OR "FokI" OR "rs1544410" OR "BsmI" OR "rs7975232" OR "ApaI" OR "rs731236" OR "TaqI")))  ("hypertension"[MeSH Terms] OR ("Arterial Hypertension"[All Fields] OR "Essential Hypertension"[All Fields])) AND ("vitamin d"[MeSH Terms] OR "ergocalciferols"[MeSH Terms] OR ("cholecalciferol"[MeSH Terms] OR "cholecalciferol"[All Fields] OR "cholecalciferols"[All Fields] OR "colecalciferol"[All Fields] OR ("ergocalciferols"[MeSH Terms] OR "ergocalciferols"[All Fields] OR "ergocalciferol"[All Fields]))) AND ("polymorphism, genetic"[MeSH Terms] OR "polymorphism, single nucleotide"[MeSH Terms] OR ("Gene Variation"[All Fields] OR "Genetic Variation"[All Fields] OR "Polymorphism"[All Fields] OR ("socioaffect neurosci psychol"[Journal] OR "snp"[All Fields]) OR ((("rs2228570"[All Fields] OR "FokI"[All Fields] OR "rs1544410"[All Fields] OR "BsmI"[All Fields] OR "rs7975232"[All Fields] OR "ApaI"[All Fields] OR "rs731236"[All Fields]) OR "TaqI"[All Fields]))) | 30 |
| ProQuest | noft(Hypertension) AND noft("Vitamin D" OR Cholecalciferol OR Ergocalciferol) AND noft("Gene Polymorphism" OR "Single Nucleotide Polymorphism" OR "Gene Variation" OR "Genetic Variation" OR "Polymorphism" OR SNP OR "rs2228570" OR "FokI" OR "rs1544410" OR "BsmI" OR "rs7975232" OR "ApaI" OR "rs731236" OR "TaqI") | 88 |
| EBSCO | (AB hypertension OR TI (hypertension)) AND (AB ( "Vitamin D" OR Cholecalciferol OR Ergocalciferol ) OR ( TI "Vitamin D" OR Cholecalciferol OR Ergocalciferol )) AND (AB ( "Gene Polymorphism" OR "Single Nucleotide Polymorphism" OR "Gene Variation" OR "Genetic Variation" OR "Polymorphism" OR SNP ) OR TI ( "Gene Polymorphism" OR "Single Nucleotide Polymorphism" OR "Gene Variation" OR "Genetic Variation" OR "Polymorphism" OR SNP ) OR AB ("rs2228570" OR "FokI" OR "rs1544410" OR "BsmI" OR "rs7975232" OR "ApaI" OR "rs731236" OR "TaqI") OR TI ("rs2228570" OR "FokI" OR "rs1544410" OR "BsmI" OR "rs7975232" OR "ApaI" OR "rs731236" OR "TaqI")) | 112 |
| Cochrane Library | (Hypertension) AND ("Vitamin D" OR Cholecalciferol OR Ergocalciferol) AND ("Gene Polymorphism" OR "Single Nucleotide Polymorphism" OR "Gene Variation" OR "Genetic Variation" OR "Polymorphism" OR SNP OR "rs2228570" OR "FokI" OR "rs1544410" OR "BsmI" OR "rs7975232" OR "ApaI" OR "rs731236" OR "TaqI") | 9 |
| Science Direct | (Hypertension) AND ("Vitamin D") AND ("Gene Polymorphism" OR "Single Nucleotide Polymorphism" OR "Gene Variation" OR "Genetic Variation" OR "Polymorphism" OR SNP OR "rs2228570" OR "FokI" OR "rs1544410" OR "BsmI" OR "rs7975232" OR "ApaI" OR "rs731236" OR "TaqI") within Title, Abstract, Keyword | 23 |
| Springer Link | (Hypertension) AND ("Vitamin D") AND ("Gene Polymorphism" OR "Single Nucleotide Polymorphism" OR "Gene Variation" OR "Genetic Variation" OR "Polymorphism" OR SNP OR "rs2228570" OR "FokI" OR "rs1544410" OR "BsmI" OR "rs7975232" OR "ApaI" OR "rs731236" OR "TaqI") within Article Content Type | 2007 |
| EMBASE | hypertension:ti,ab,kw AND ('vitamin d':ti,ab,kw OR cholecalciferol:ti,ab,kw OR ergocalciferol:ti,ab,kw) AND ('gene polymorphism':ti,ab,kw OR 'single nucleotide polymorphism':ti,ab,kw OR 'gene variation':ti,ab,kw OR 'genetic variation':ti,ab,kw OR 'polymorphism':ti,ab,kw OR snp:ti,ab,kw 'rs2228570':ti,ab,kw OR 'FokI':ti,ab,kw OR 'rs1544410':ti,ab,kw OR 'BsmI':ti,ab,kw OR 'rs7975232':ti,ab,kw OR 'ApaI':ti,ab,kw OR 'rs731236':ti,ab,kw OR 'TaqI':ti,ab,kw) | 97 |
| LILACS | (Hypertension) AND ("Vitamin D") AND ("Gene Polymorphism" OR "Single Nucleotide Polymorphism" OR "Gene Variation" OR "Genetic Variation" OR "Polymorphism" OR SNP OR "rs2228570" OR "FokI" OR "rs1544410" OR "BsmI" OR "rs7975232" OR "ApaI" OR "rs731236" OR "TaqI") within Title, Abstract, Keyword | 2 |

**Supplementary Table S1.** Keywords Used in Database Searching
