# Supplementary material for: Association between vitamin D receptor gene polymorphism and essential hypertension: An updated systematic review, meta-analysis, and meta-regression
Source: PLoS One. 2024 Dec 23;19(12):e0314886. doi: 10.1371/journal.pone.0314886 (PMC11666036; doi:10.1371/journal.pone.0314886)
Supplement: S2 Table — (DOCX) [file pone.0314886.s002.docx]

**Supplementary Table S2.** Newcastle Ottawa Scale for Case-Control Studies

| Study | Selection | | | | | Comparability | Exposure | | | | Overall Total |
| --- | --- | --- | --- | --- | --- | --- | --- | --- | --- | --- | --- |
|  | Case definition adequate or not | Representativeness of the cases | Selection of controls | Definition of controls | Subtotal |  | Ascertainment of exposure | Same method of ascertainment for cases and controls | Non-response rate | Subtotal | Total / 9 |
| Swapna et al., 2011 | 1 | 0 | 0 | 1 | 2 | 0 | 1 | 1 | 0 | 2 | 4 |
| Vural et al., 2012 | 1 | 0 | 0 | 1 | 2 | 0 | 1 | 1 | 0 | 2 | 4 |
| Glocke et al., 2013 | 1 | 0 | 1 | 1 | 3 | 0 | 1 | 1 | 0 | 2 | 5 |
| Errouagui et al., 2014 | 1 | 0 | 1 | 1 | 3 | 0 | 1 | 1 | 0 | 2 | 5 |
| Jia et al, 2014 | 1 | 1 | 1 | 1 | 4 | 1 | 1 | 1 | 0 | 2 | 7 |
| Cottone et al., 2015 | 1 | 0 | 0 | 1 | 2 | 0 | 1 | 1 | 0 | 2 | 4 |
| Gussago et al., 2016 | 1 | 1 | 1 | 1 | 4 | 0 | 1 | 1 | 0 | 2 | 6 |
| Lai et al., 2016 | 1 | 1 | 1 | 1 | 3 | 2 | 1 | 1 | 0 | 2 | 8 |
| Ye et al., 2018 | 1 | 0 | 1 | 1 | 3 | 2 | 1 | 1 | 0 | 2 | 7 |
| Zhang et al., 2018 | 1 | 1 | 1 | 1 | 3 | 2 | 1 | 1 | 0 | 2 | 8 |
| Xia et al., 2019 | 1 | 1 | 1 | 1 | 3 | 2 | 1 | 1 | 0 | 2 | 8 |
| Obukhova et al., 2020 | 1 | 0 | 0 | 1 | 2 | 1 | 1 | 1 | 0 | 2 | 5 |
| Varakantham et al., 2020 | 1 | 0 | 1 | 1 | 3 | 1 | 1 | 1 | 0 | 2 | 6 |
| Prasad et al., 2021 | 1 | 0 | 0 | 1 | 2 | 0 | 1 | 1 | 0 | 2 | 4 |
| Repchuk et al., 2021 | 1 | 0 | 0 | 1 | 2 | 0 | 1 | 1 | 0 | 2 | 4 |
| Zehra et al., 2022 | 1 | 0 | 0 | 1 | 2 | 1 | 1 | 1 | 0 | 2 | 5 |
| Gariballa et al., 2023 | 1 | 0 | 1 | 1 | 3 | 2 | 1 | 1 | 0 | 2 | 7 |
| Rojo-Tolosa et al., 2023 | 1 | 1 | 1 | 1 | 3 | 2 | 1 | 1 | 0 | 2 | 8 |
| Nabil et al., 2024 | 1 | 1 | 1 | 1 | 3 | 2 | 1 | 1 | 0 | 2 | 8 |

**Swapna et al., 2011** = no defined period of time for cases representatives, no description for source of controls included, the sex and age matched control was statement only but the evidence for these adjustment were not clarified, no description on non-response rate

**Vural et al., 2012** = no defined period of time for cases representatives, no description for source of controls included, the sex and age matched control was statement only but the evidence for these adjustment were not clarified, no description on non-response rate

**Glocke et al., 2013** = no defined period of time for cases representatives, no adjustment made for age and other factors, no description on non-response rate

**Errouagui et al., 2014** = no defined period of time for cases representatives, the sex matched control was statement only but the evidence for this adjustment was not clarified and there was no adjustment for age, no description on non-response rate

**Jia et al, 2014** = Adjustment was only made for sex but not age, no description on non-response rate

**Cottone et al., 2015** = no defined catchment area and period of time for cases representatives, no description for source of controls included, the sex and age matched control was statement only but the evidence for these adjustment were not clarified, no description on non-response rate

**Gussago et al., 2016** = no adjustment made for age and other factors, no description on non-response rate

**Lai et al., 2016 =** no description on non-response rate

**Ye et al., 2018 =** no defined period of time for cases representatives, no description on non-response rate

**Zhang et al., 2018** = no description on non-response rate

**Xia et al., 2019 =** no description on non-response rate

**Obukhova et al., 2020** = no defined catchment area and period of time for cases representatives, adjustment was only made for sex but not age, no description on non-response rate

**Varakantham et al., 2020 =** No defined period of time for cases representatives, adjustment was only made for sex but not age, no description on non-response rate

**Prasad et al., 2021 =** no defined period of time for cases representatives, no description for source of controls included, the sex and age matched control was statement only but the evidence for these adjustment were not clarified, no description on non-response rate

**Repchuk et al., 2021** = no defined catchment area and period of time for cases representatives, the sex and age matched control was statement only but the evidence for these adjustment were not clarified, no description on non-response rate

**Zehra et al., 2022 =** no defined catchment area and period of time for cases representatives, no description for source of controls included, the sex matched control was statement only but the evidence for this adjustment was not clarified, no description on non-response rate

**Gariballa et al., 2023** = no defined period of time for cases representatives, no description on non-response rate

**Rojo-Tolosa et al., 2023** = no description on non-response rate

**Nabil et al., 2024** = no description on non-response rate
