# Supplementary material for: Association between vitamin D receptor gene polymorphism and essential hypertension: An updated systematic review, meta-analysis, and meta-regression
Source: PLoS One. 2024 Dec 23;19(12):e0314886. doi: 10.1371/journal.pone.0314886 (PMC11666036; doi:10.1371/journal.pone.0314886)
Supplement: S3 Table — (DOCX) [file pone.0314886.s003.docx]

**Supplementary Table S3.** Newcastle Ottawa Scale for Cross-Sectional Studies

| Study | Selection | | | | | Comparability | | Outcome | | | | Overall Total |
| --- | --- | --- | --- | --- | --- | --- | --- | --- | --- | --- | --- | --- |
|  | Sample represents target population | Sample size adequate or justified | Non-respondents | Ascertainment of the exposure (risk factor) | Subtotal | The study control for the most important factor | The study control for any additional factor | Outcome assessment | Statistical test | Subtotal | | Total / 10 |
| Vaidya et al., 2011 | 1 | 1 | 1 | 2 | 5 | 1 | 1 | 2 | 1 | 3 | 10 | |
| Santos et al., 2021 | 1 | 0 | 1 | 2 | 4 | 1 | 1 | 2 | 1 | 3 | 9 | |

**Santos et al., 2021 =** There was no further information for justification whether sample size was adequate
