# Supplementary material for: Association between vitamin D receptor gene polymorphism and essential hypertension: An updated systematic review, meta-analysis, and meta-regression
Source: PLoS One. 2024 Dec 23;19(12):e0314886. doi: 10.1371/journal.pone.0314886 (PMC11666036; doi:10.1371/journal.pone.0314886)
Supplement: S4 Table — (DOCX) [file pone.0314886.s004.docx]

**Supplementary Table S4.** Newcastle Ottawa Scale for Cohort Studies

| Study | Selection | | | | | Comparability | Exposure | | | | Overall Total |
| --- | --- | --- | --- | --- | --- | --- | --- | --- | --- | --- | --- |
|  | Representativeness of the exposed cohort | Selection of the non exposed cohort | Ascertainment of exposure | No presence of outcome | Subtotal |  | Ascertainment of outcome | Follow-up duration | Adequacy of follow up of cohorts | Subtotal | Total / 9 |
| Wang et al., 2013 | 1 | 1 | 1 | 1 | 4 | 2 | 0 | 1 | 1 | 2 | 8 |

**Wang et al., 2013** = Outcome ascertainment was based on self report and questionairre
