# Supplementary figures and images for: Association between vitamin D receptor gene polymorphism and essential hypertension: An updated systematic review, meta-analysis, and meta-regression
Source: PLoS One. 2024 Dec 23;19(12):e0314886. doi: 10.1371/journal.pone.0314886 (PMC11666036; doi:10.1371/journal.pone.0314886)

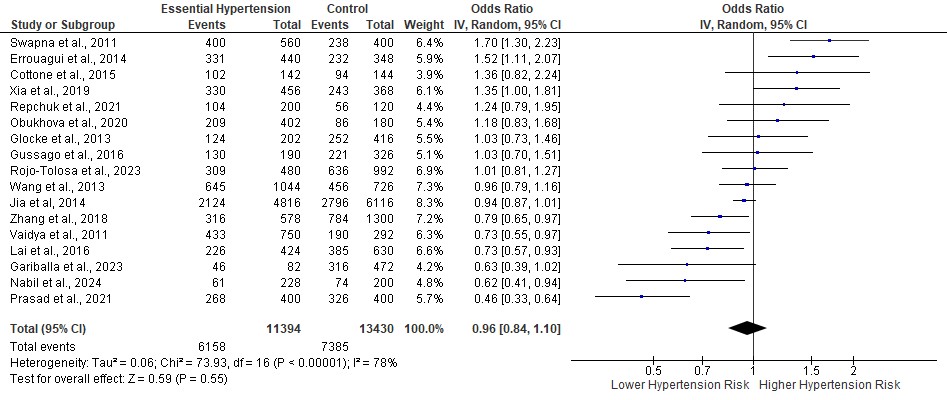

Supplement: S1 Fig — (JPG) [file pone.0314886.s005.jpg]

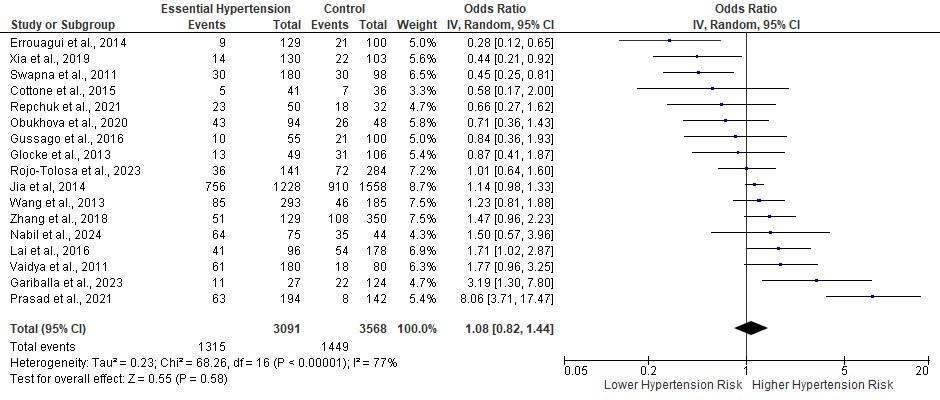

Supplement: S2 Fig — (JPG) [file pone.0314886.s006.jpg]

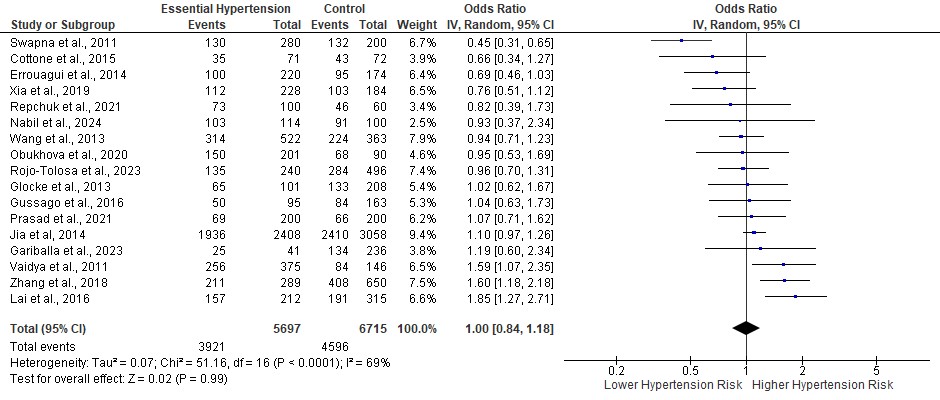

Supplement: S3 Fig — (JPG) [file pone.0314886.s007.jpg]

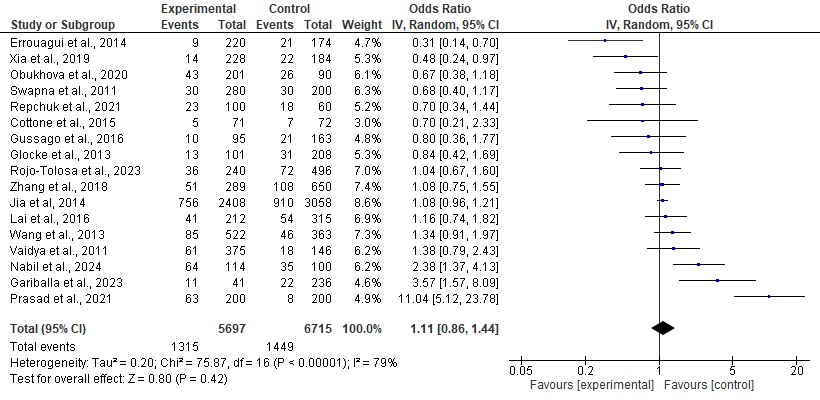

Supplement: S4 Fig — (JPG) [file pone.0314886.s008.jpg]

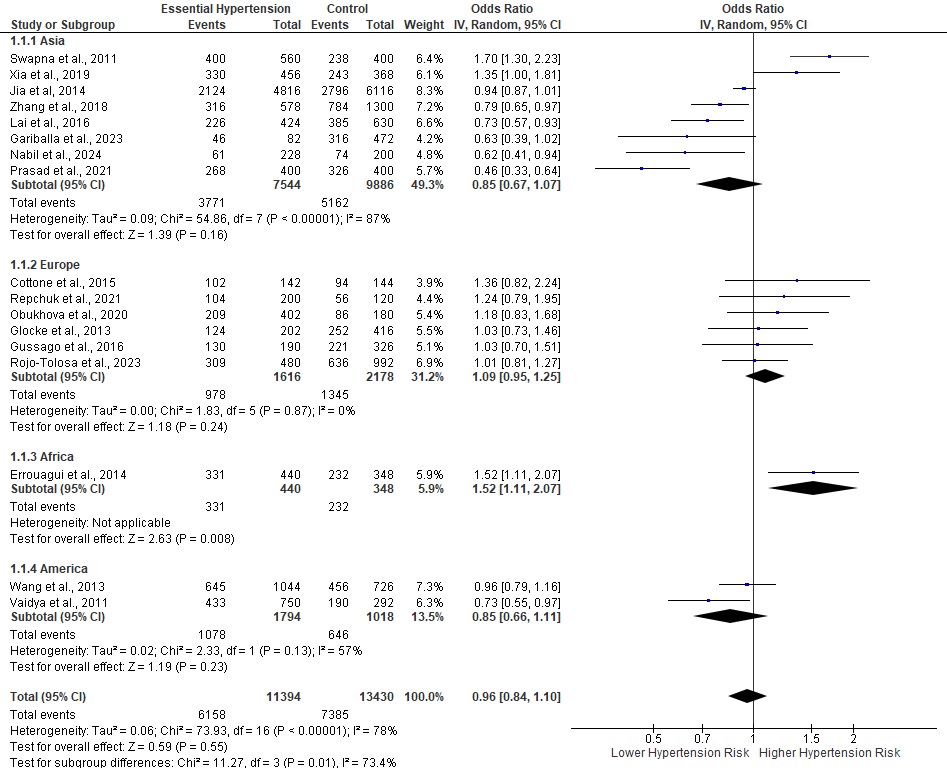

Supplement: S5 Fig — (JPG) [file pone.0314886.s009.jpg]

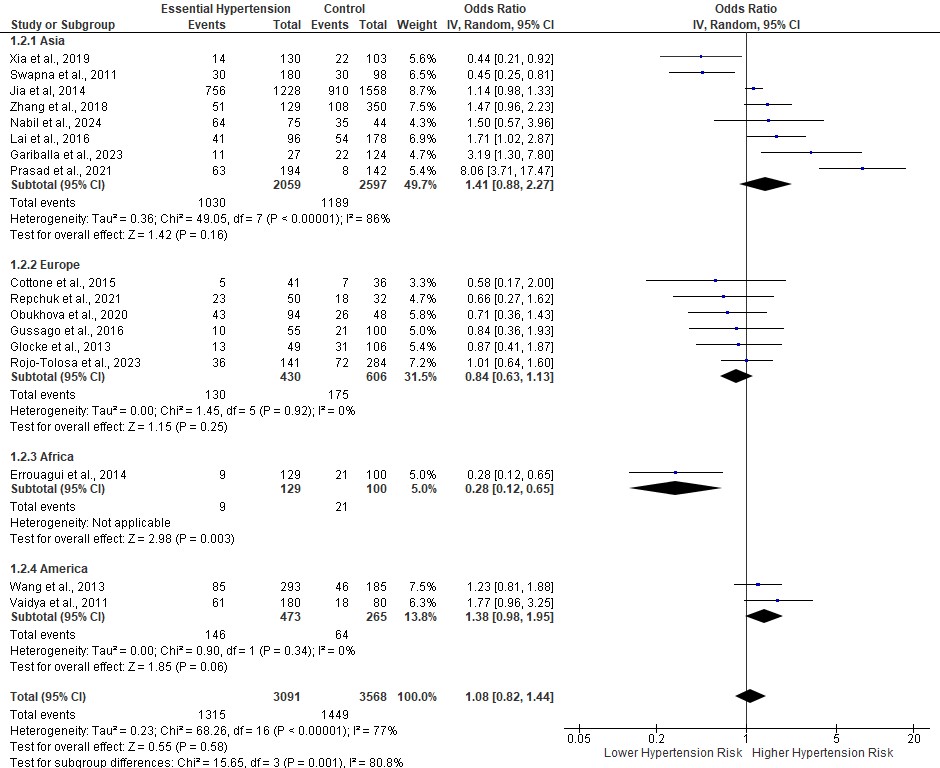

Supplement: S6 Fig — (JPG) [file pone.0314886.s010.jpg]

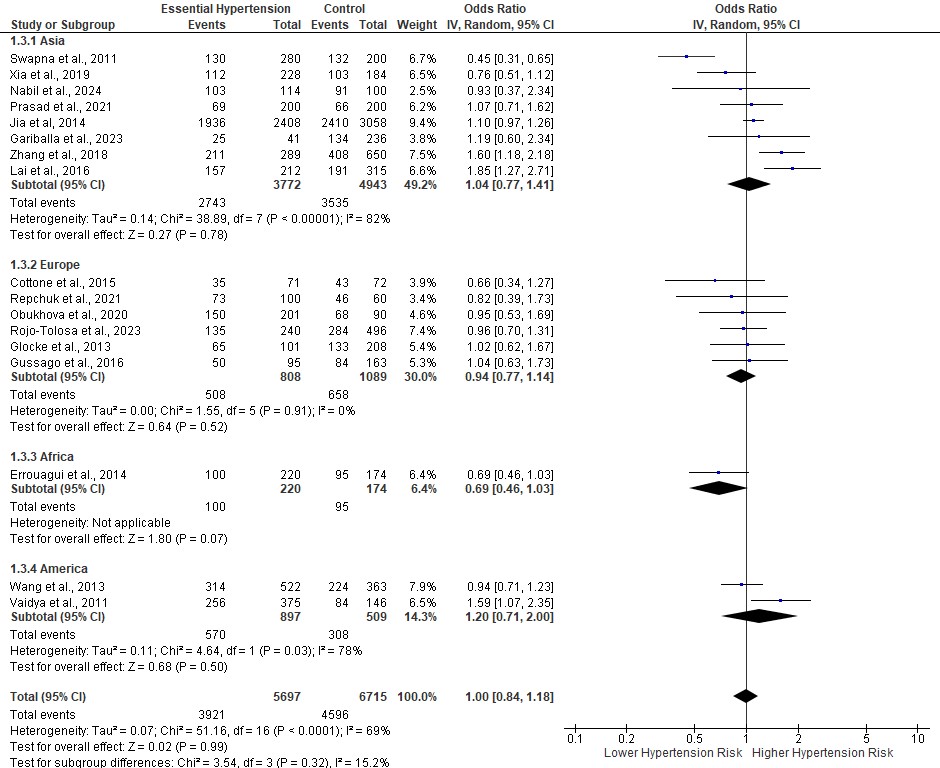

Supplement: S7 Fig — (JPG) [file pone.0314886.s011.jpg]

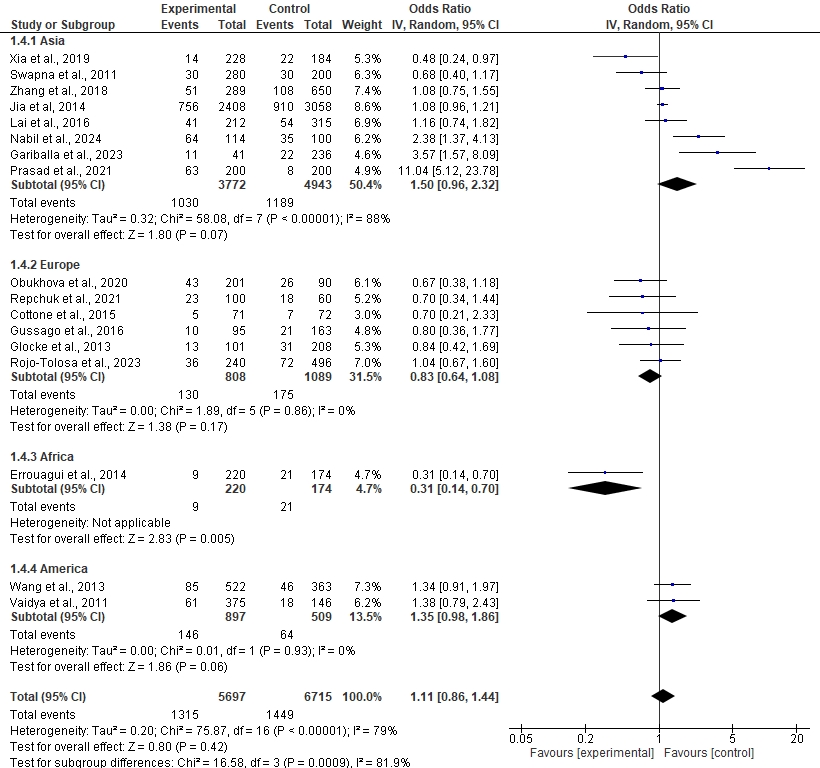

Supplement: S8 Fig — (JPG) [file pone.0314886.s012.jpg]

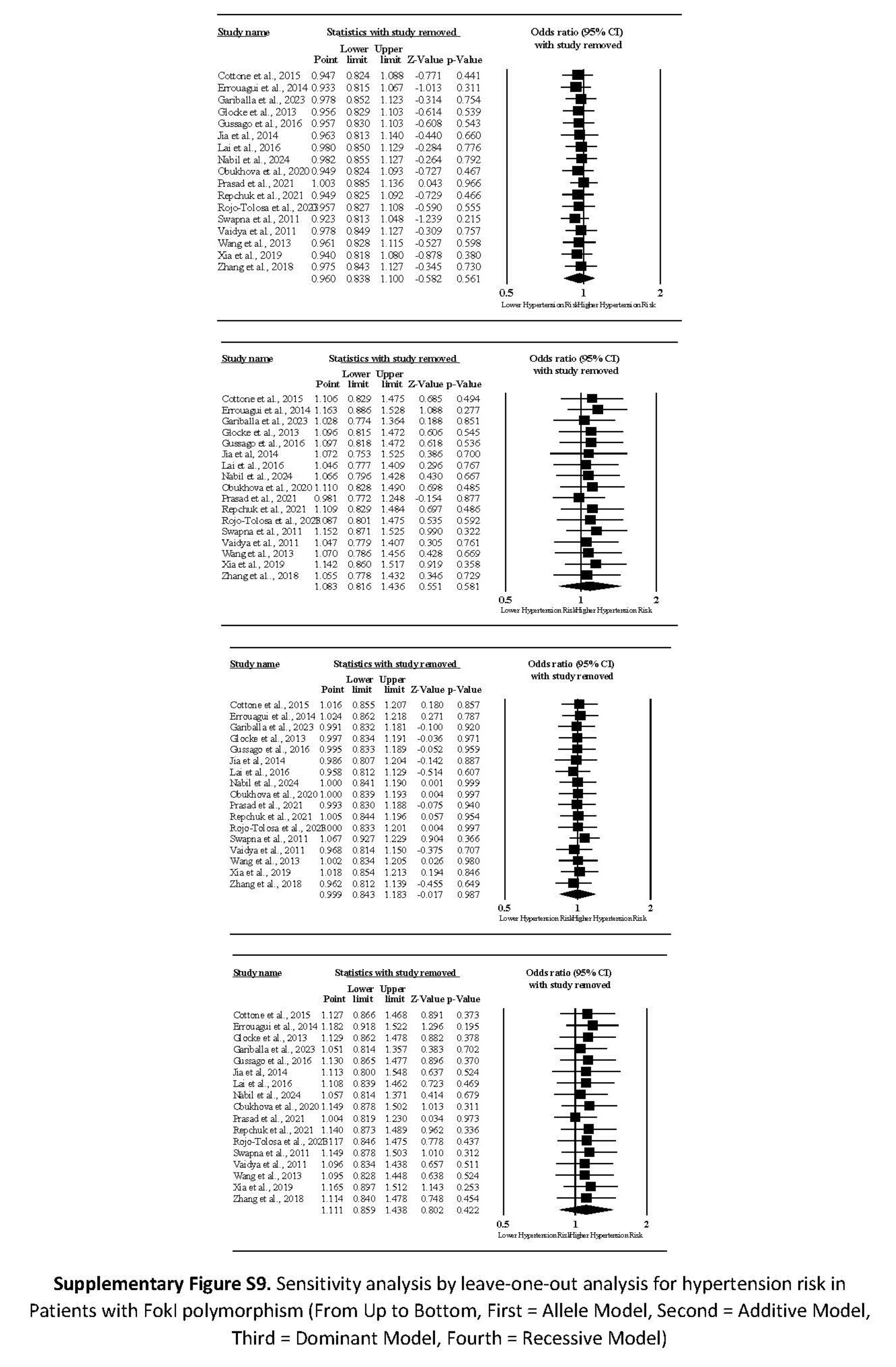

Supplement: S9 Fig — Sensitivity analysis by leave-one-out analysis for hypertension risk in Patients with FokI polymorphism (From Up to Bottom, First = Allele Model, Second = Additive Model, Third = Dominant Model, Fourth = Recessive Model). (JPG) [file pone.0314886.s013.jpg]

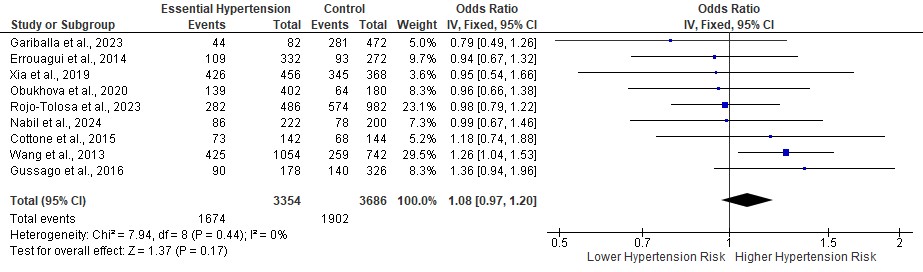

Supplement: S10 Fig — (JPG) [file pone.0314886.s014.jpg]

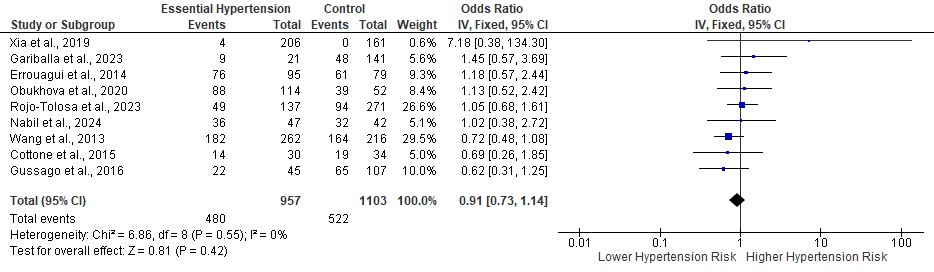

Supplement: S11 Fig — (JPG) [file pone.0314886.s015.jpg]

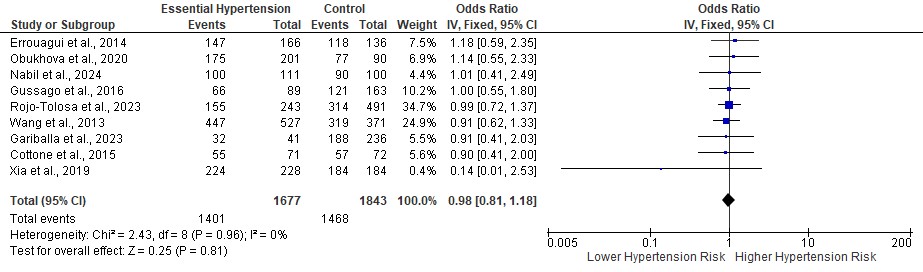

Supplement: S12 Fig — (JPG) [file pone.0314886.s016.jpg]

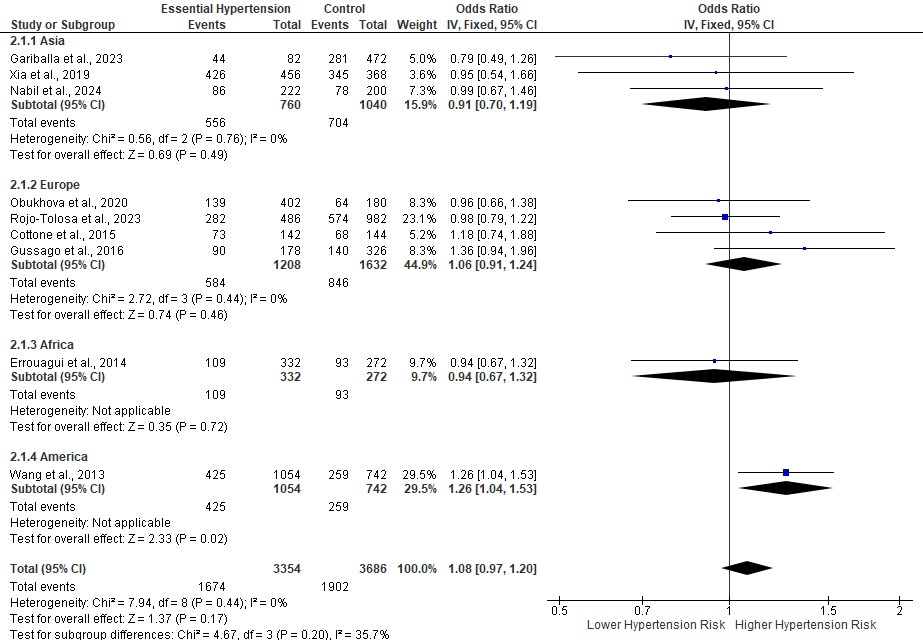

Supplement: S13 Fig — (JPG) [file pone.0314886.s017.jpg]

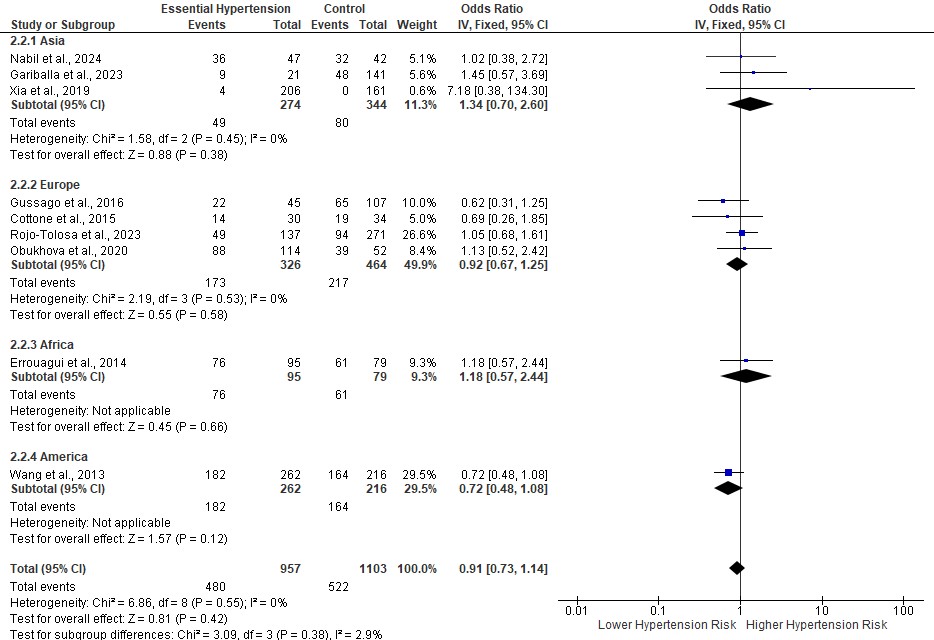

Supplement: S14 Fig — (JPG) [file pone.0314886.s018.jpg]

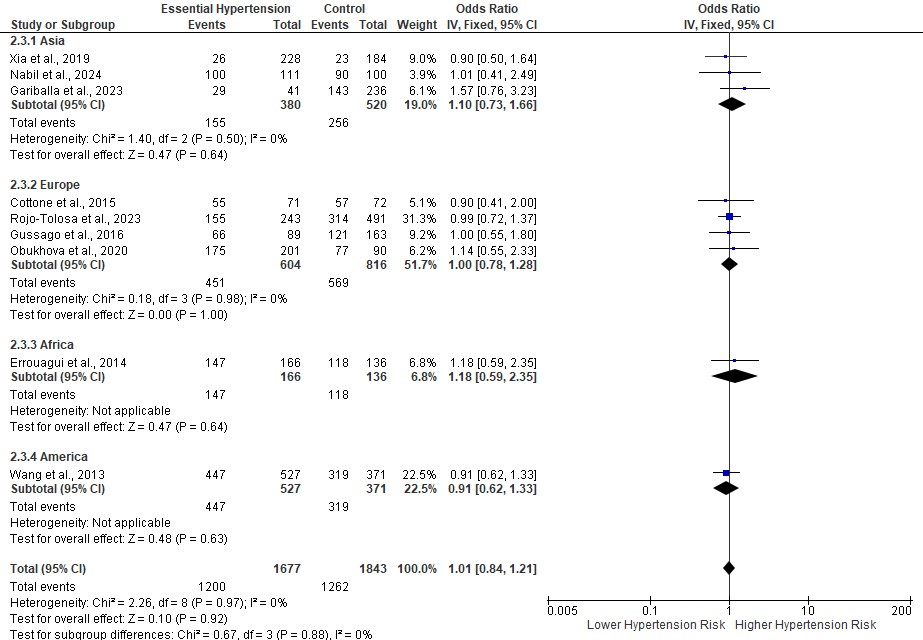

Supplement: S15 Fig — (JPG) [file pone.0314886.s019.jpg]

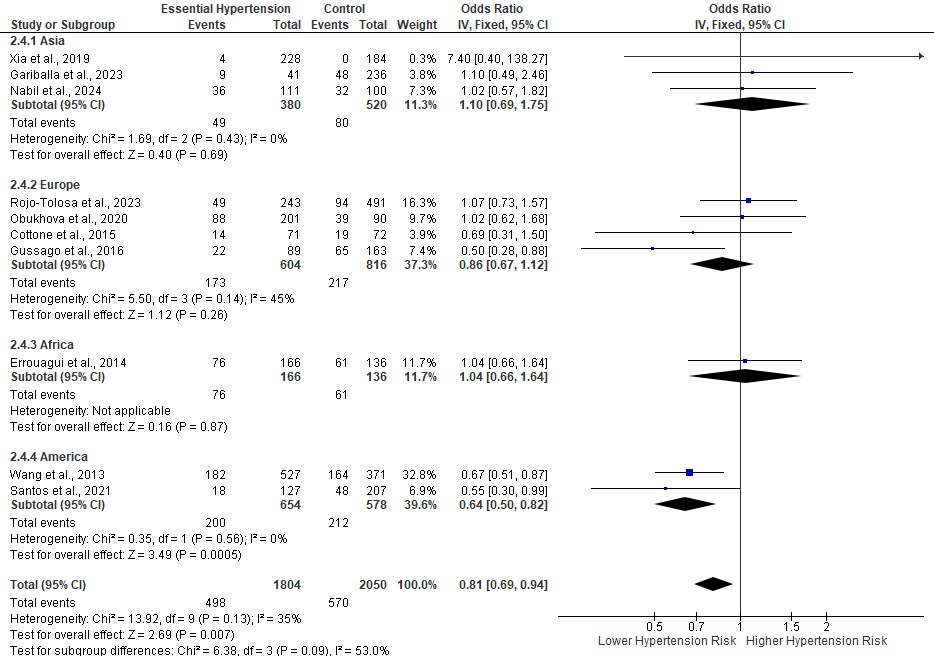

Supplement: S16 Fig — (JPG) [file pone.0314886.s020.jpg]

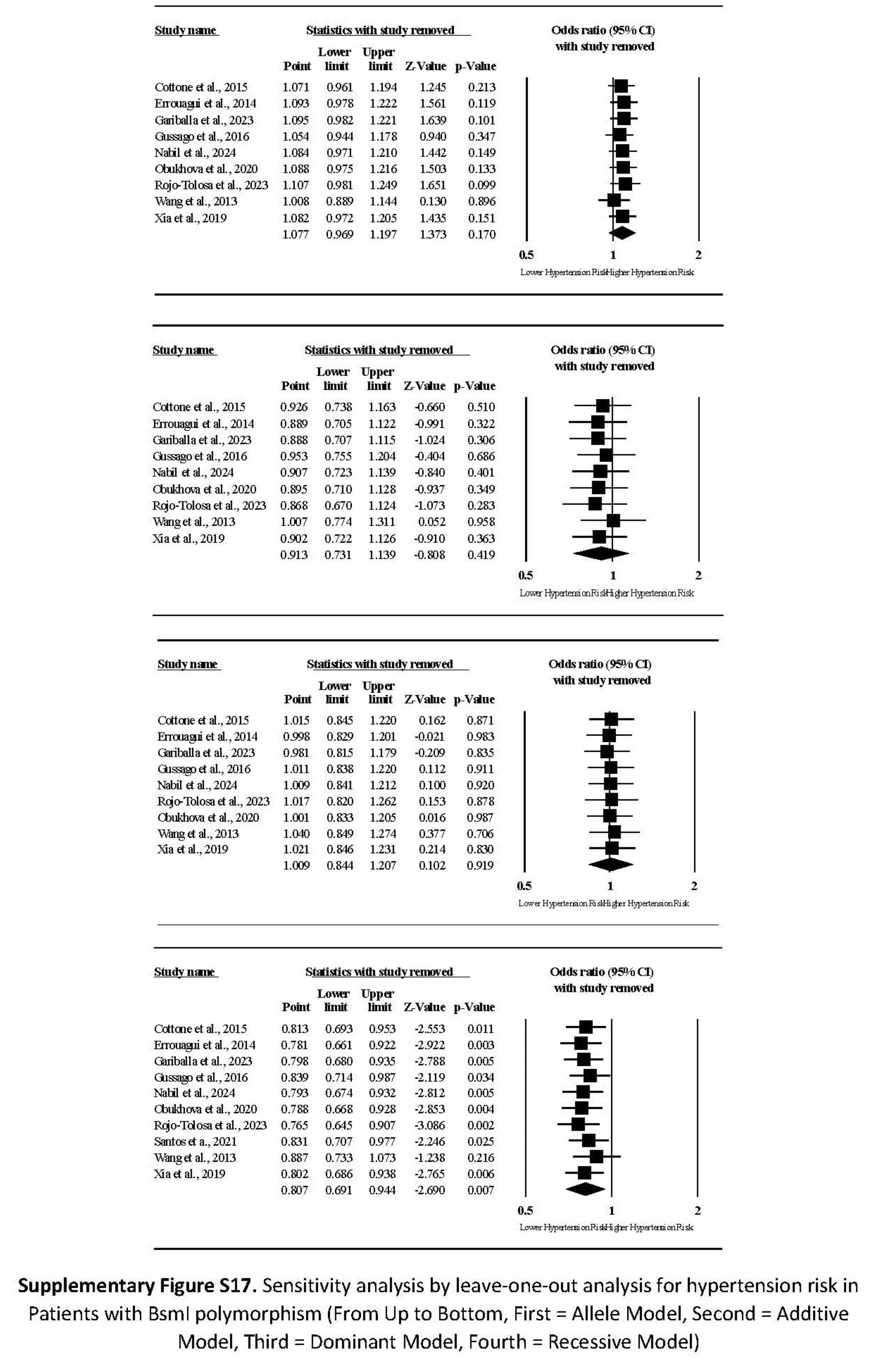

Supplement: S17 Fig — Sensitivity analysis by leave-one-out analysis for hypertension risk in Patients with BsmI polymorphism (From Up to Bottom, First = Allele Model, Second = Additive Model, Third = Dominant Model, Fourth = Recessive Model). (JPG) [file pone.0314886.s021.jpg]

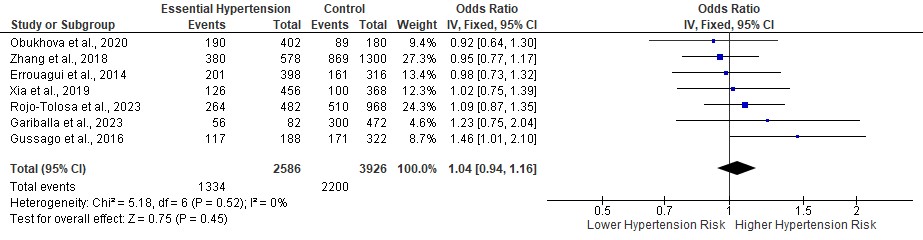

Supplement: S18 Fig — (JPG) [file pone.0314886.s022.jpg]

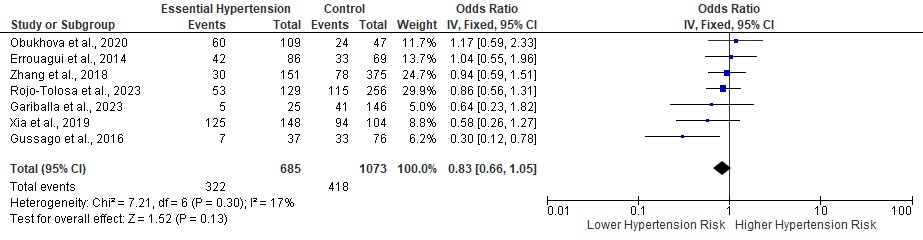

Supplement: S19 Fig — (JPG) [file pone.0314886.s023.jpg]

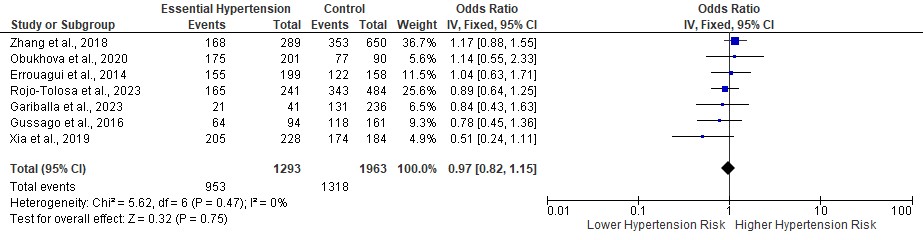

Supplement: S20 Fig — (JPG) [file pone.0314886.s024.jpg]

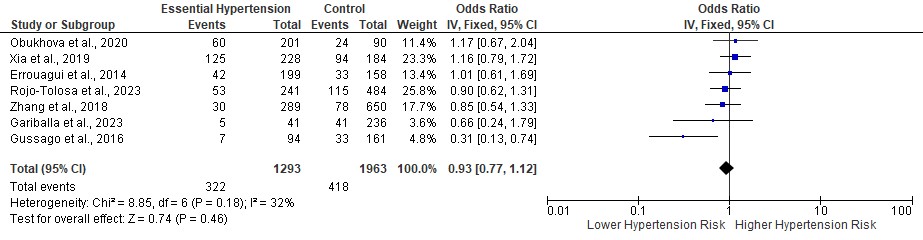

Supplement: S21 Fig — (JPG) [file pone.0314886.s025.jpg]

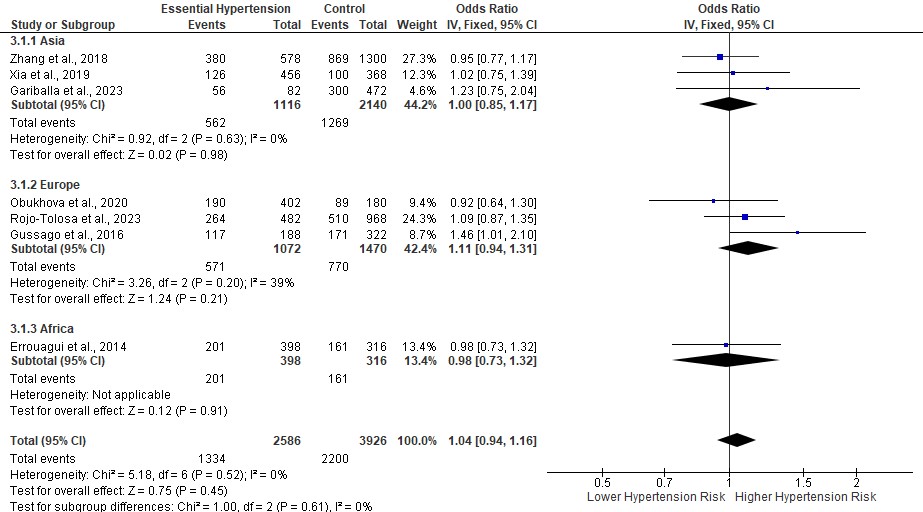

Supplement: S22 Fig — (JPG) [file pone.0314886.s026.jpg]

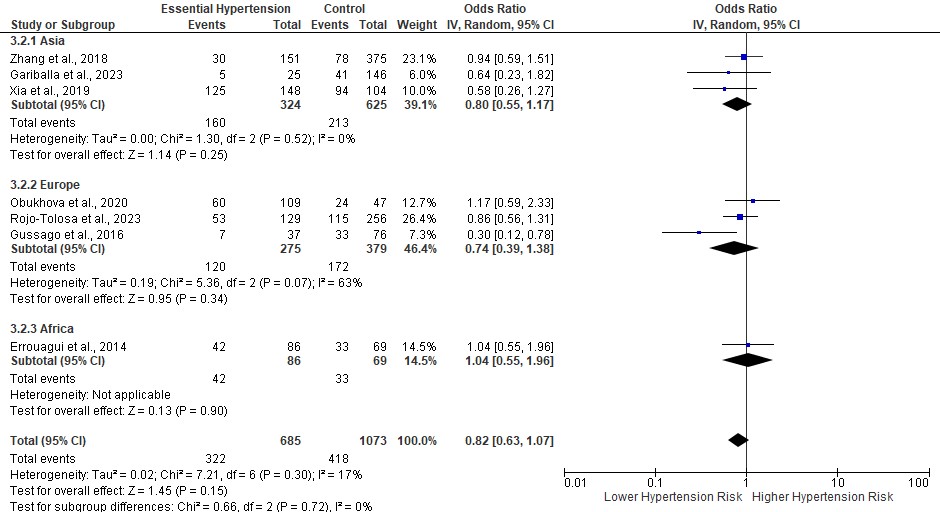

Supplement: S23 Fig — (JPG) [file pone.0314886.s027.jpg]

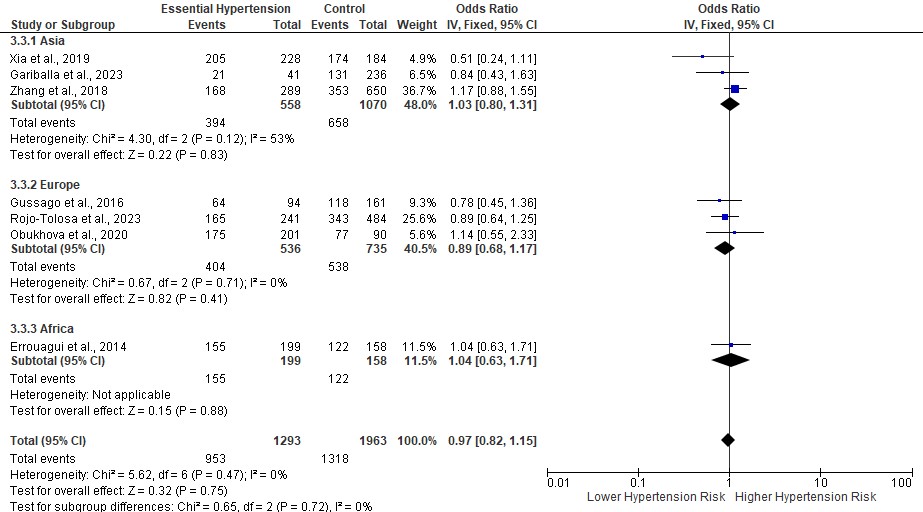

Supplement: S24 Fig — (JPG) [file pone.0314886.s028.jpg]

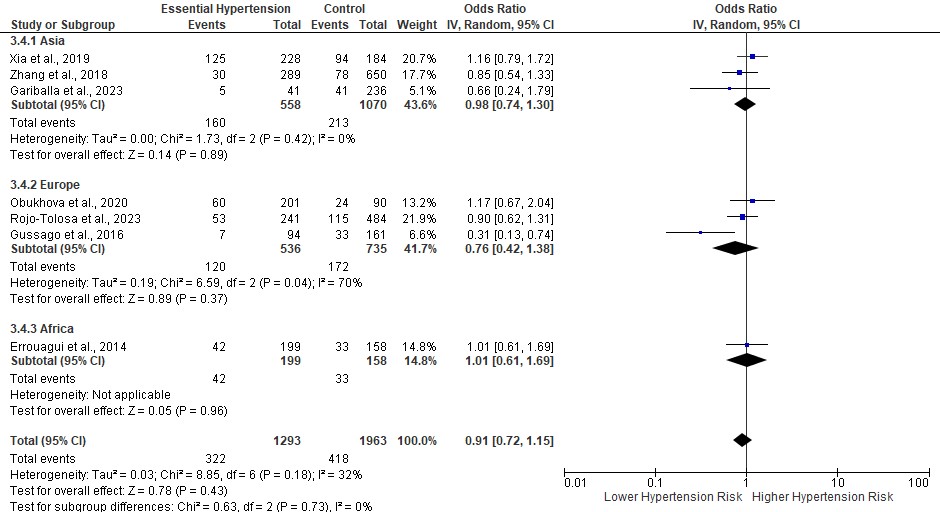

Supplement: S25 Fig — (JPG) [file pone.0314886.s029.jpg]

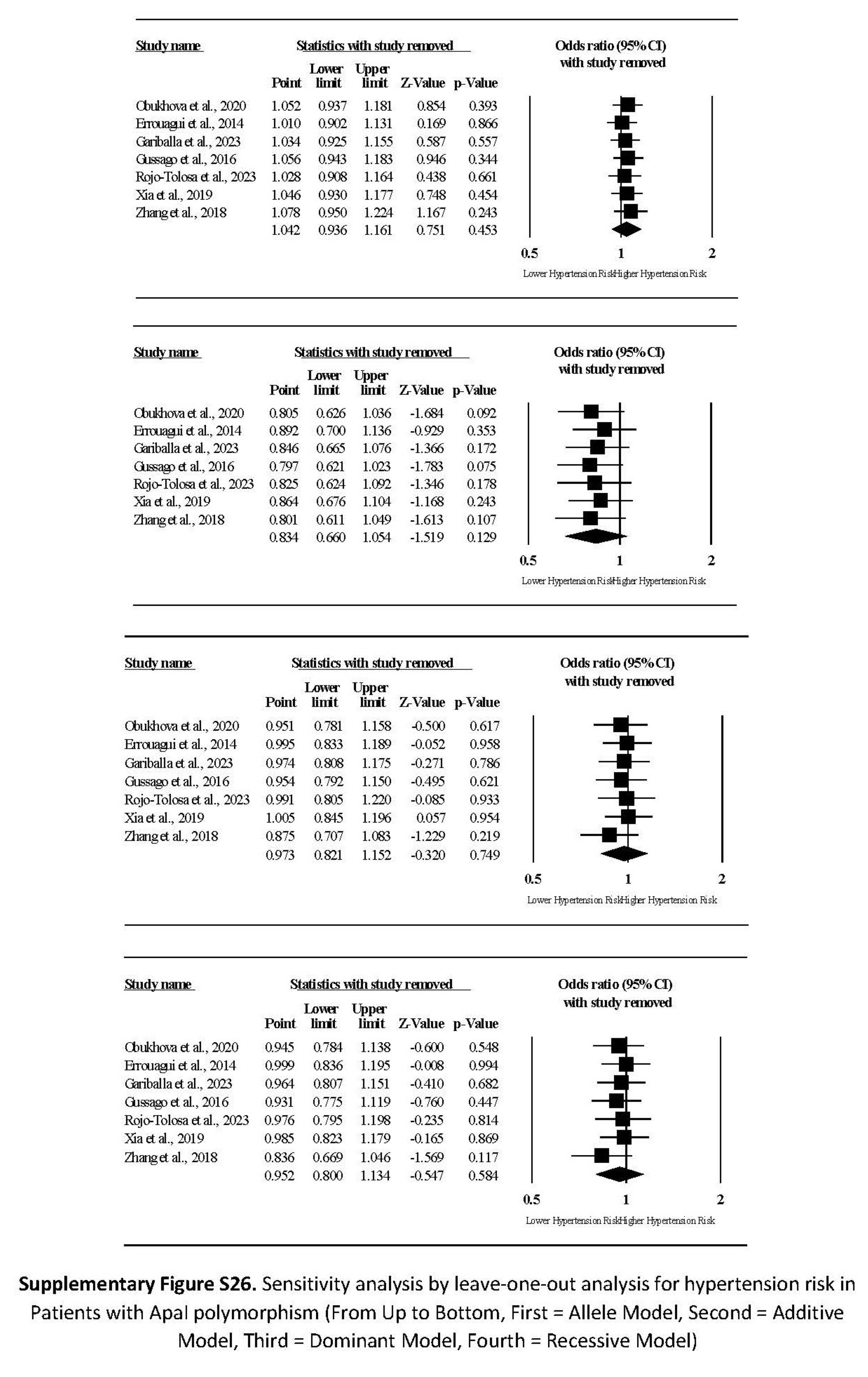

Supplement: S26 Fig — Sensitivity analysis by leave-one-out analysis for hypertension risk in Patients with ApaI polymorphism (From Up to Bottom, First = Allele Model, Second = Additive Model, Third = Dominant Model, Fourth = Recessive Model). (JPG) [file pone.0314886.s030.jpg]

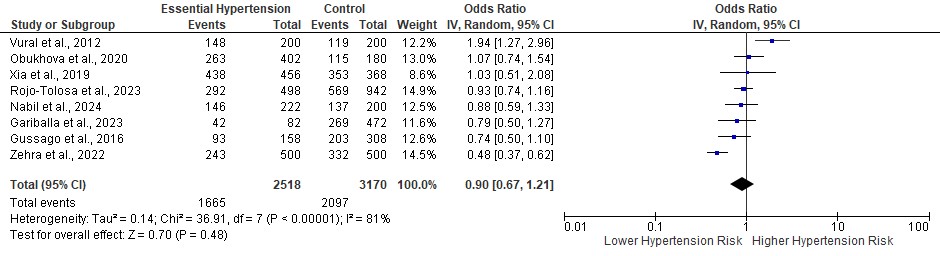

Supplement: S27 Fig — (JPG) [file pone.0314886.s031.jpg]

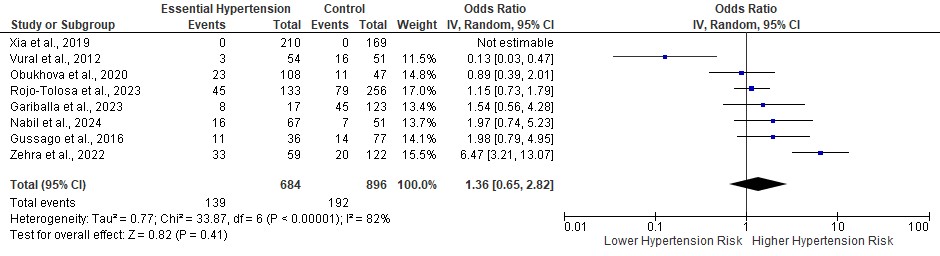

Supplement: S28 Fig — (JPG) [file pone.0314886.s032.jpg]

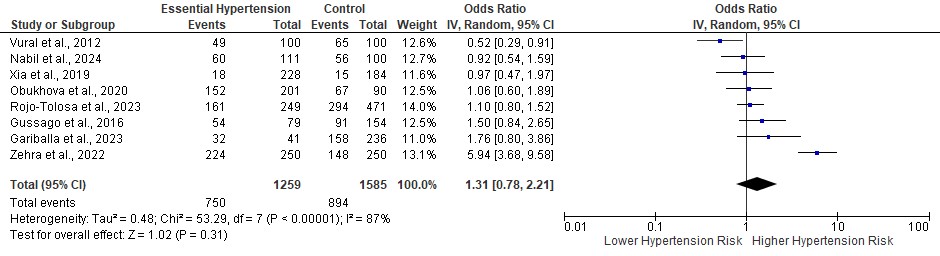

Supplement: S29 Fig — (JPG) [file pone.0314886.s033.jpg]

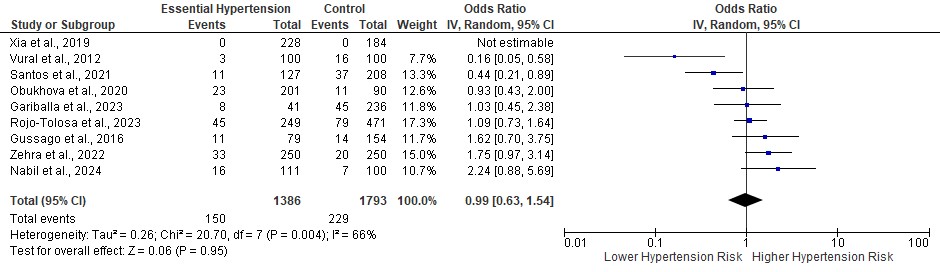

Supplement: S30 Fig — (JPG) [file pone.0314886.s034.jpg]

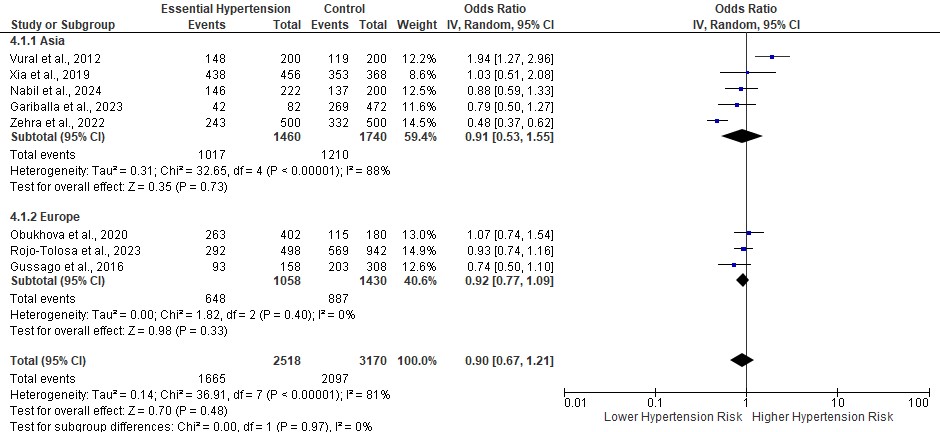

Supplement: S31 Fig — (JPG) [file pone.0314886.s035.jpg]

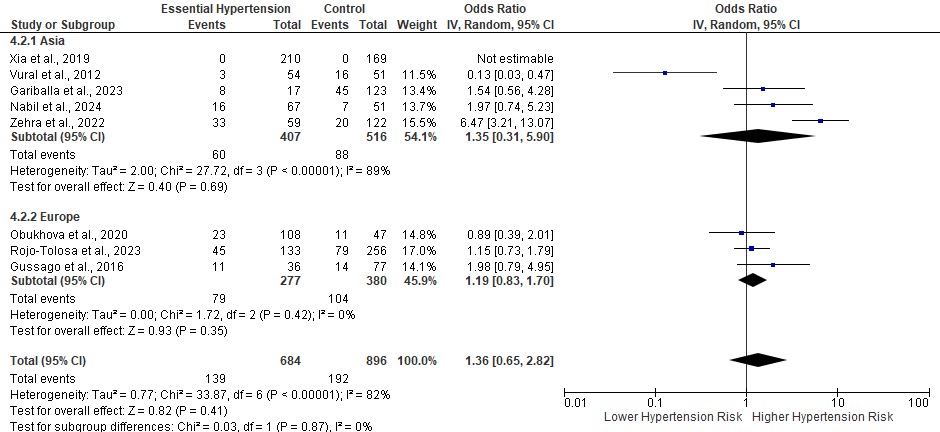

Supplement: S32 Fig — (JPG) [file pone.0314886.s036.jpg]

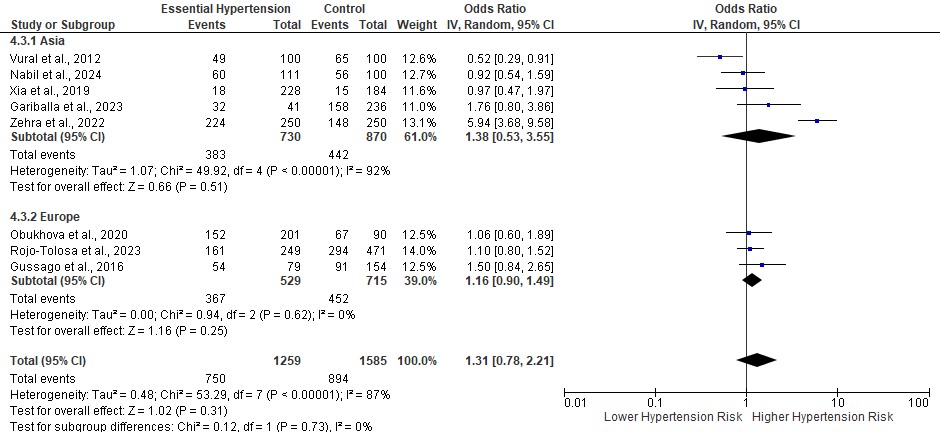

Supplement: S33 Fig — (JPG) [file pone.0314886.s037.jpg]

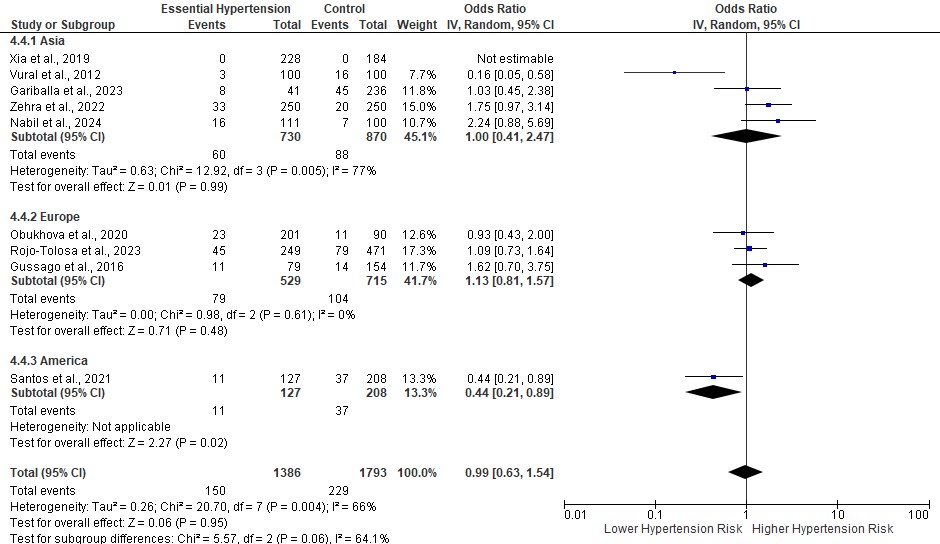

Supplement: S34 Fig — (JPG) [file pone.0314886.s038.jpg]

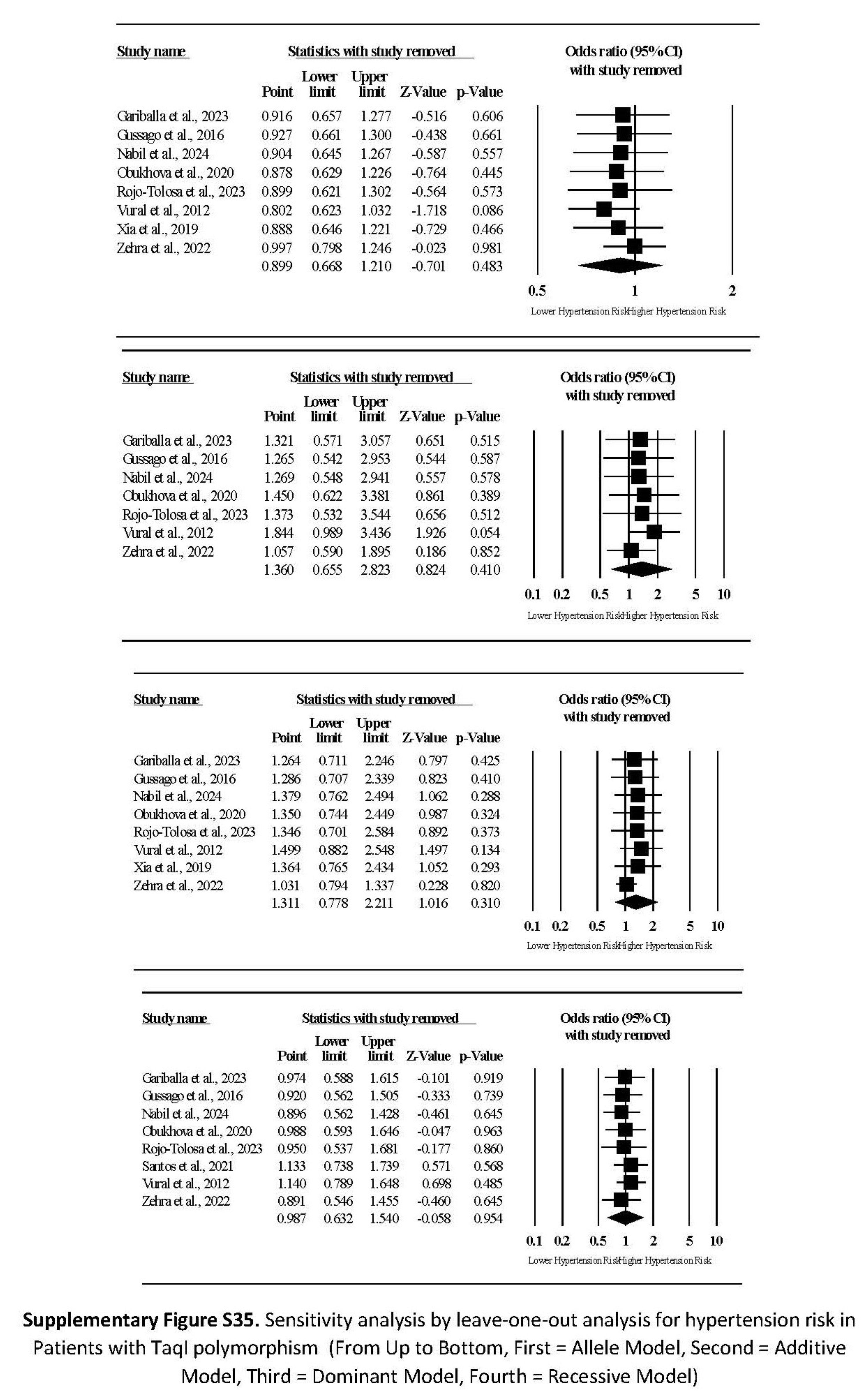

Supplement: S35 Fig — Sensitivity analysis by leave-one-out analysis for hypertension risk in Patients with TaqI polymorphism (From Up to Bottom, First = Allele Model, Second = Additive Model, Third = Dominant Model, Fourth = Recessive Model). (JPG) [file pone.0314886.s039.jpg]

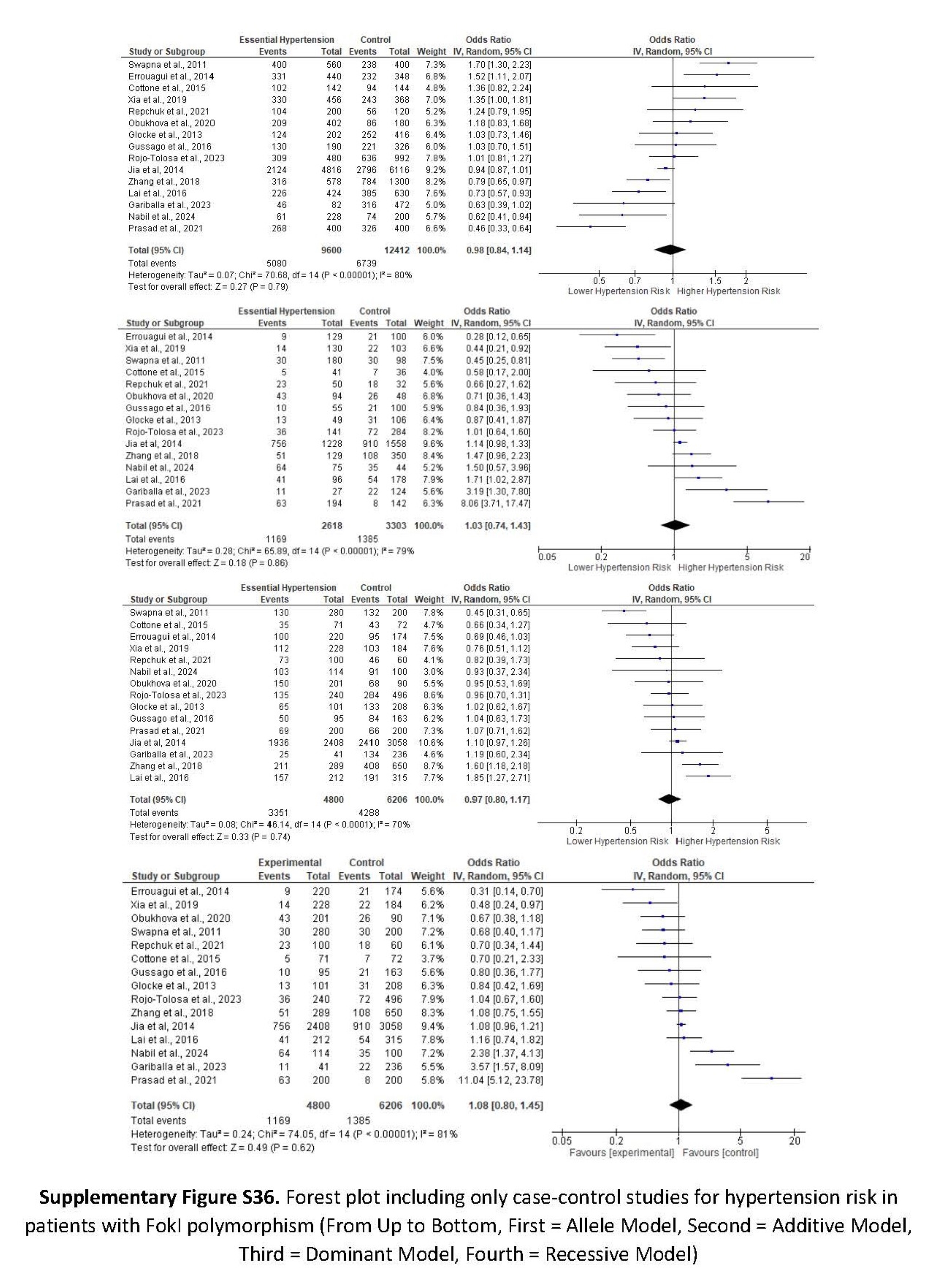

Supplement: S36 Fig — Forest plot including only case-control studies for hypertension risk in patients with FokI polymorphism (From Up to Bottom, First = Allele Model, Second = Additive Model, Third = Dominant Model, Fourth = Recessive Model). (JPG) [file pone.0314886.s040.jpg]

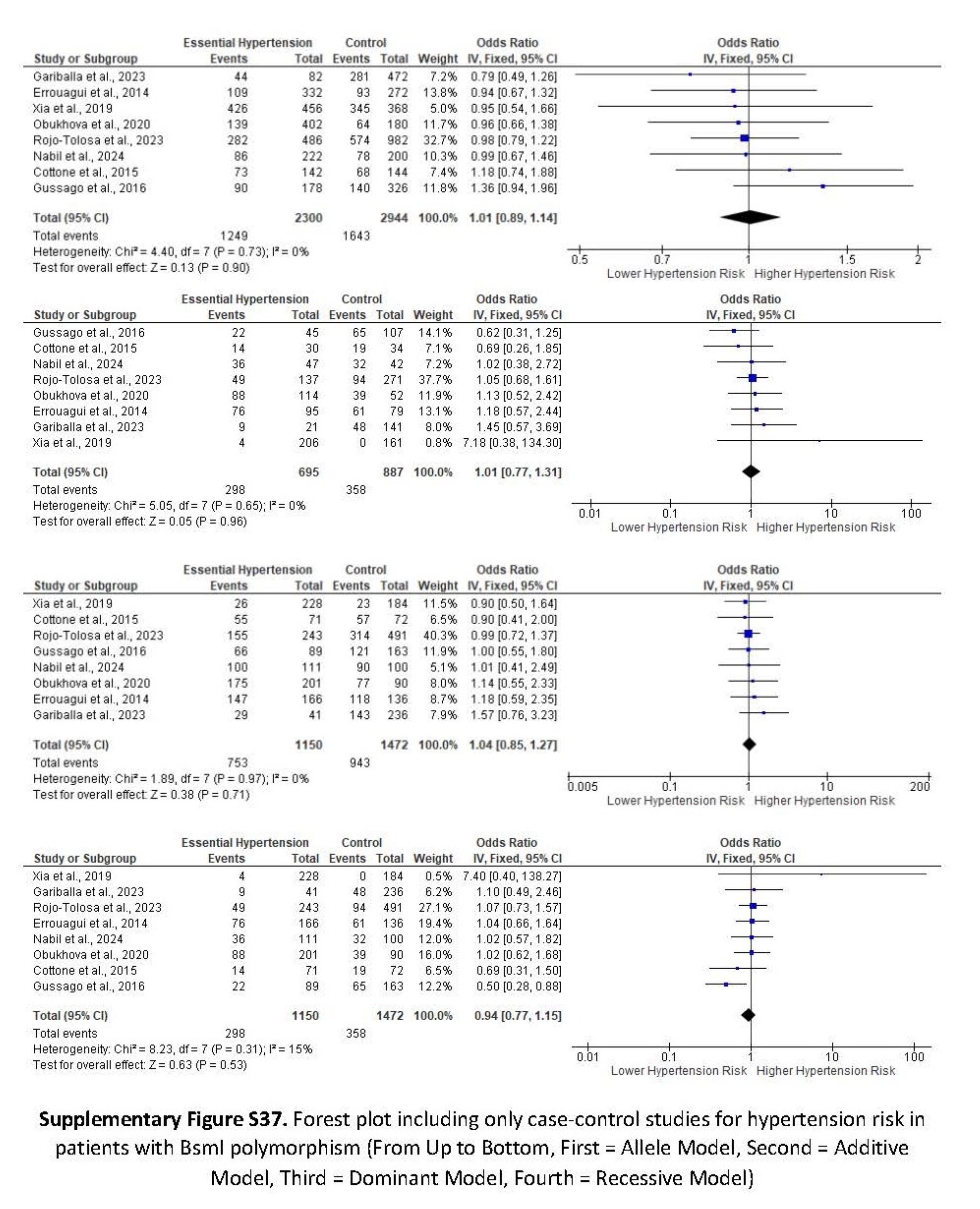

Supplement: S37 Fig — Forest plot including only case-control studies for hypertension risk in patients with BsmI polymorphism (From Up to Bottom, First = Allele Model, Second = Additive Model, Third = Dominant Model, Fourth = Recessive Model). (JPG) [file pone.0314886.s041.jpg]

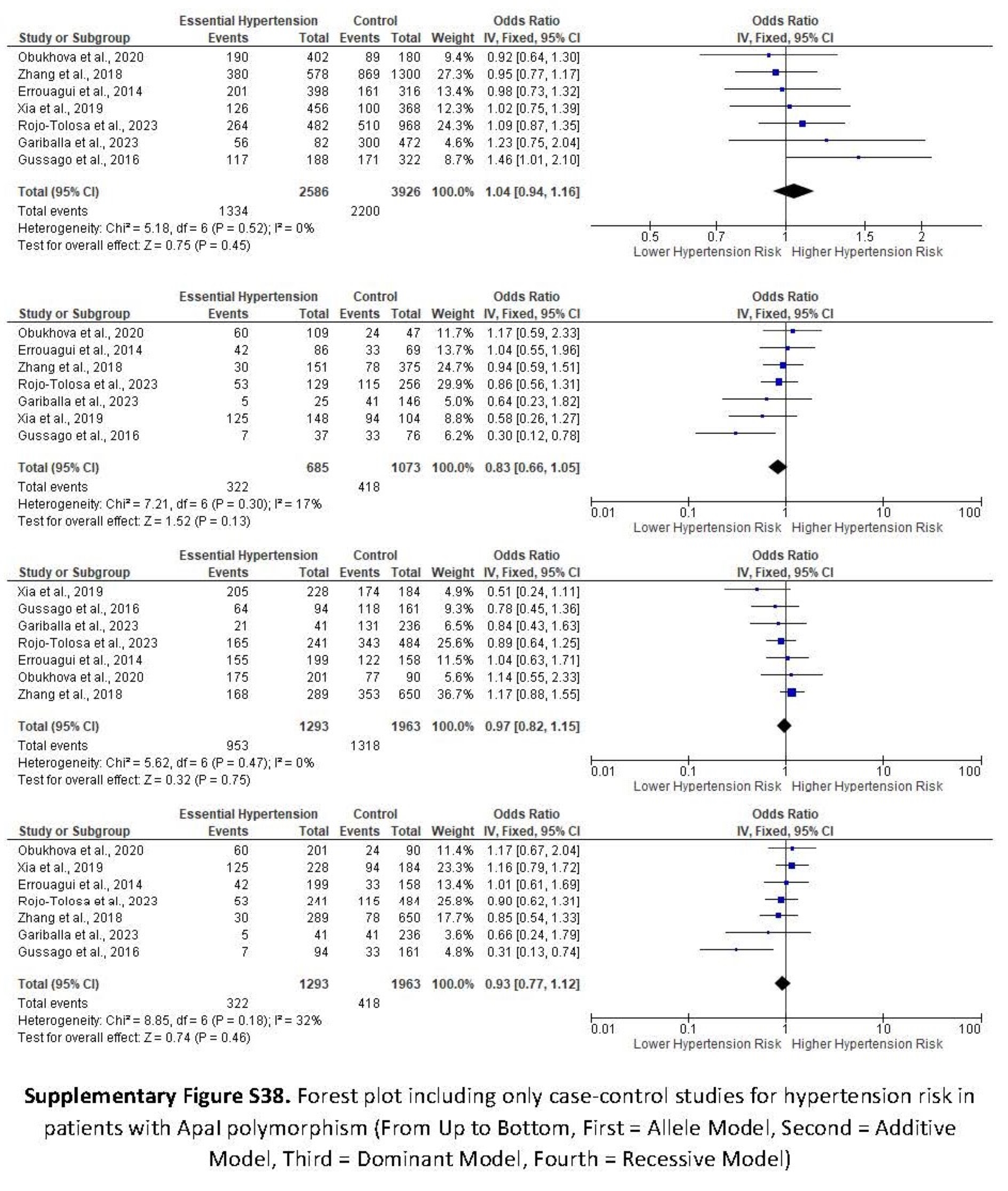

Supplement: S38 Fig — Forest plot including only case-control studies for hypertension risk in patients with ApaI polymorphism (From Up to Bottom, First = Allele Model, Second = Additive Model, Third = Dominant Model, Fourth = Recessive Model). (JPG) [file pone.0314886.s042.jpg]

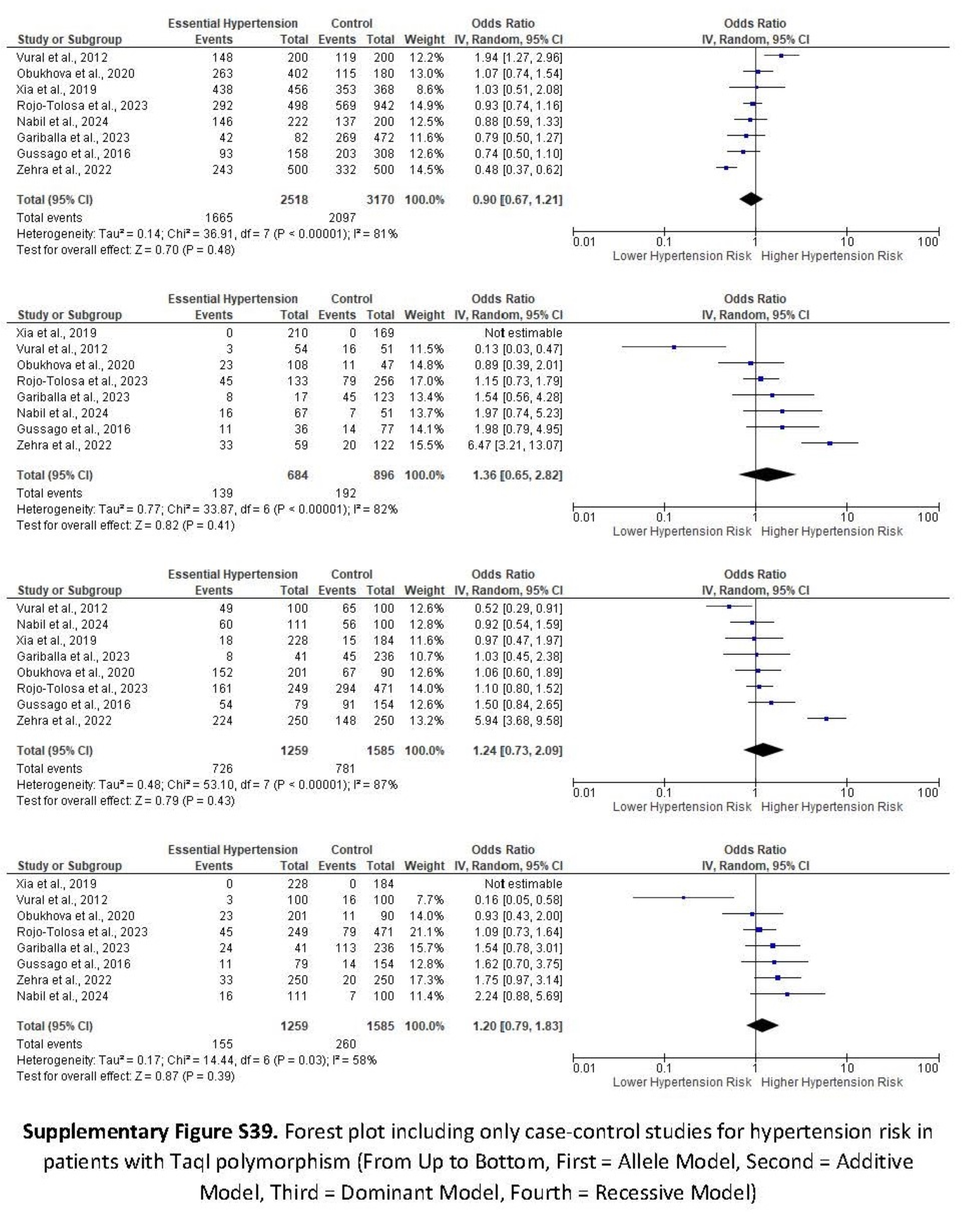

Supplement: S39 Fig — Forest plot including only case-control studies for hypertension risk in patients with TaqI polymorphism (From Up to Bottom, First = Allele Model, Second = Additive Model, Third = Dominant Model, Fourth = Recessive Model). (JPG) [file pone.0314886.s043.jpg]

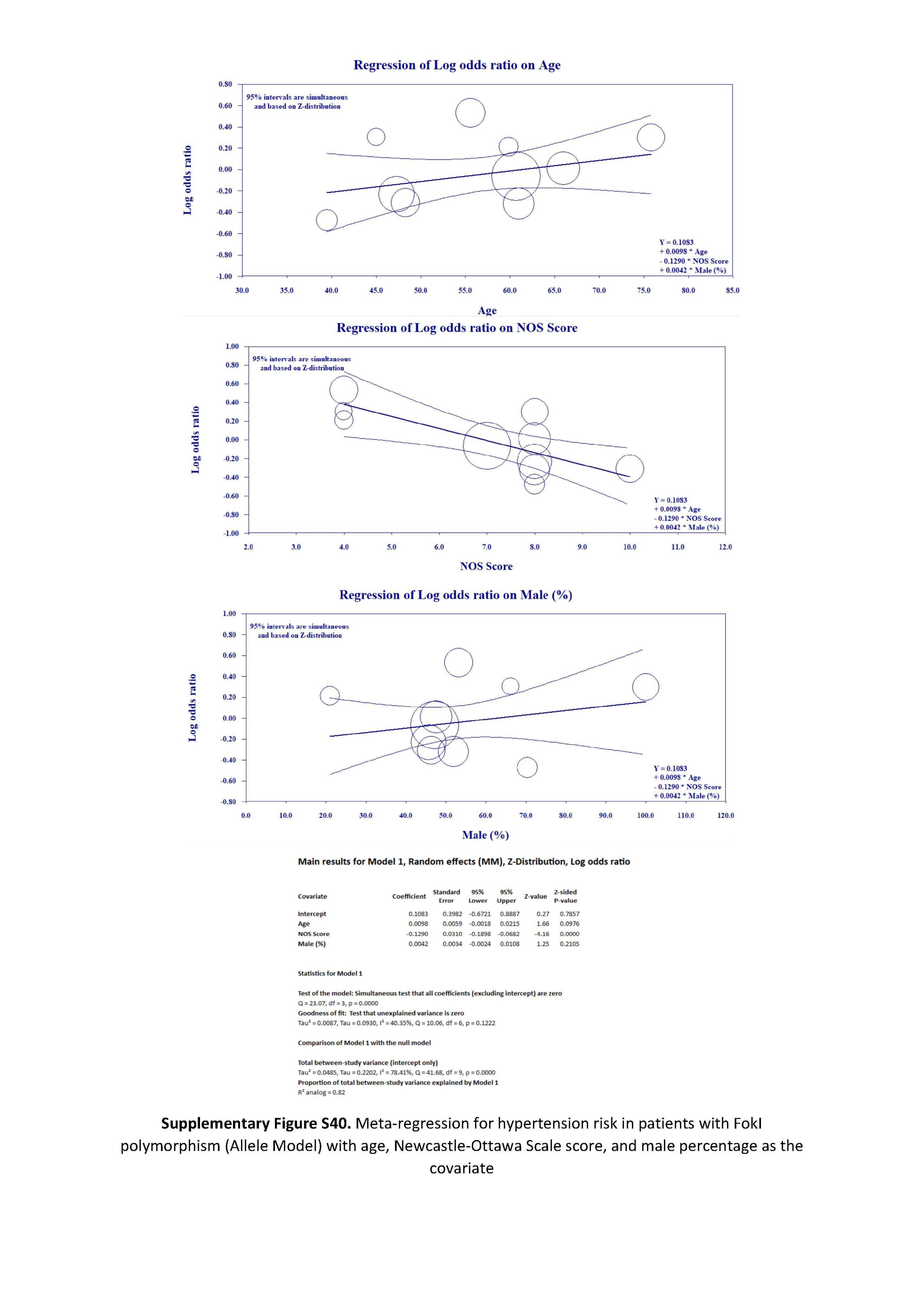

Supplement: S40 Fig — (JPG) [file pone.0314886.s044.jpg]

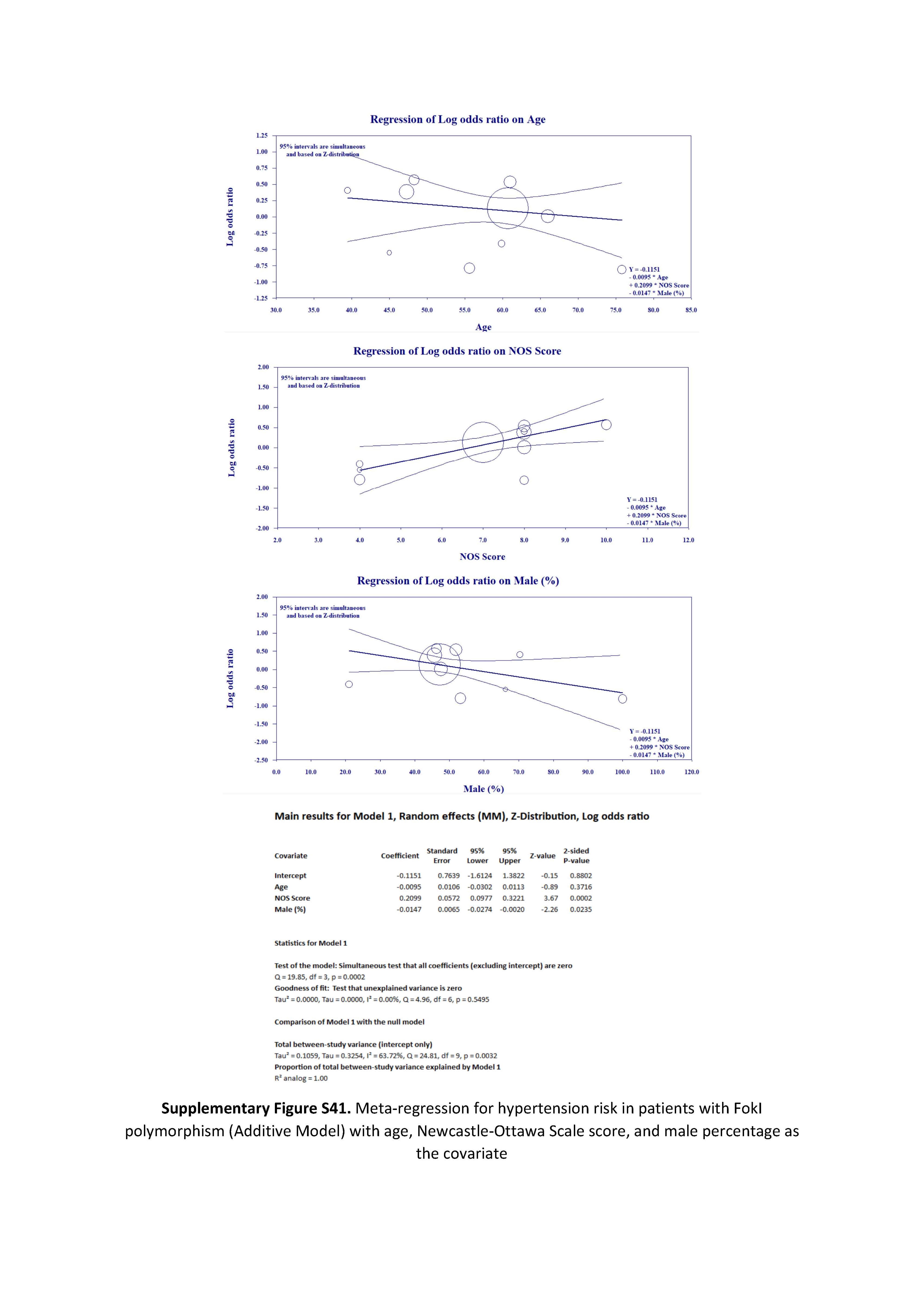

Supplement: S41 Fig — (JPG) [file pone.0314886.s045.jpg]

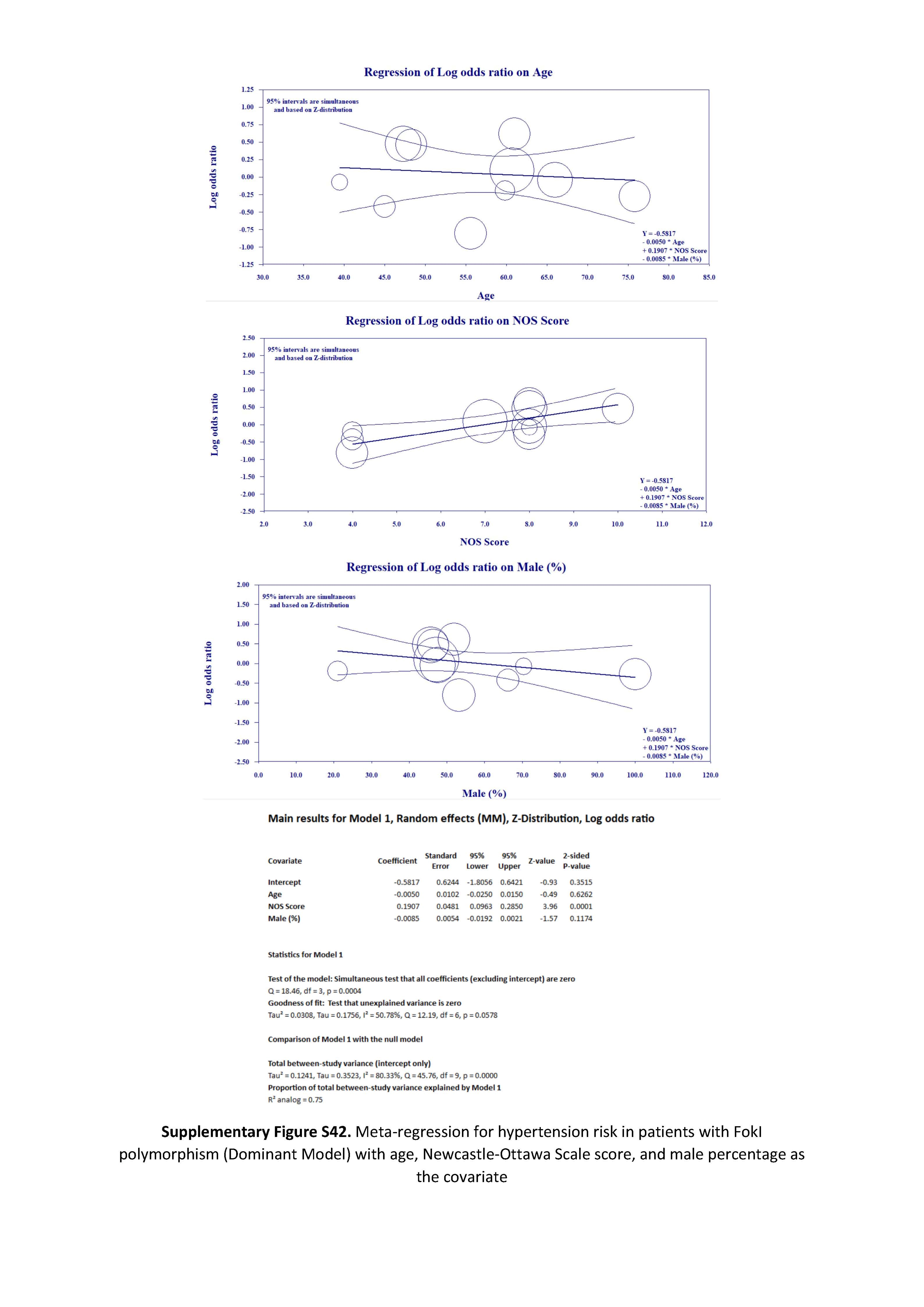

Supplement: S42 Fig — (JPG) [file pone.0314886.s046.jpg]

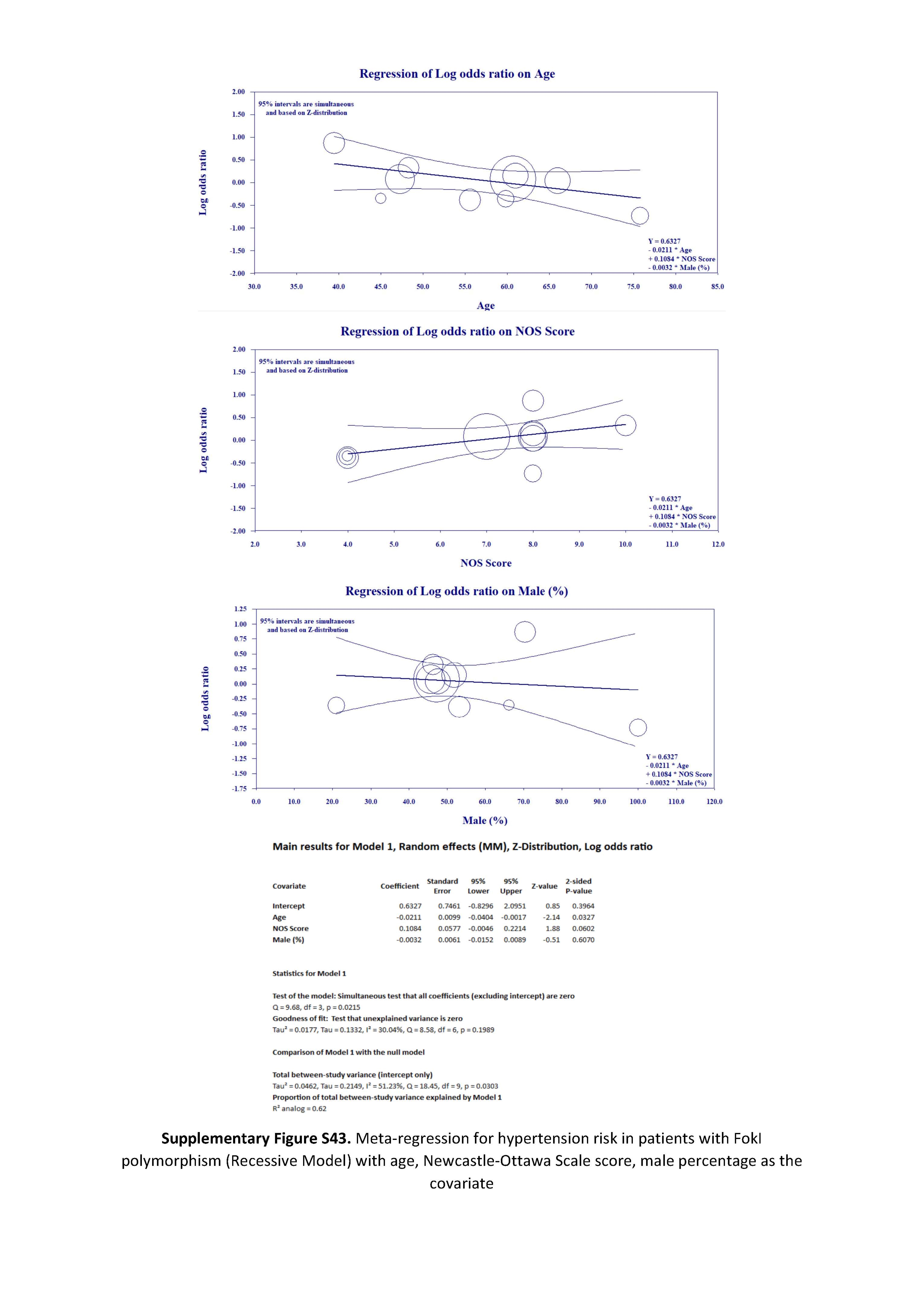

Supplement: S43 Fig — (JPG) [file pone.0314886.s047.jpg]

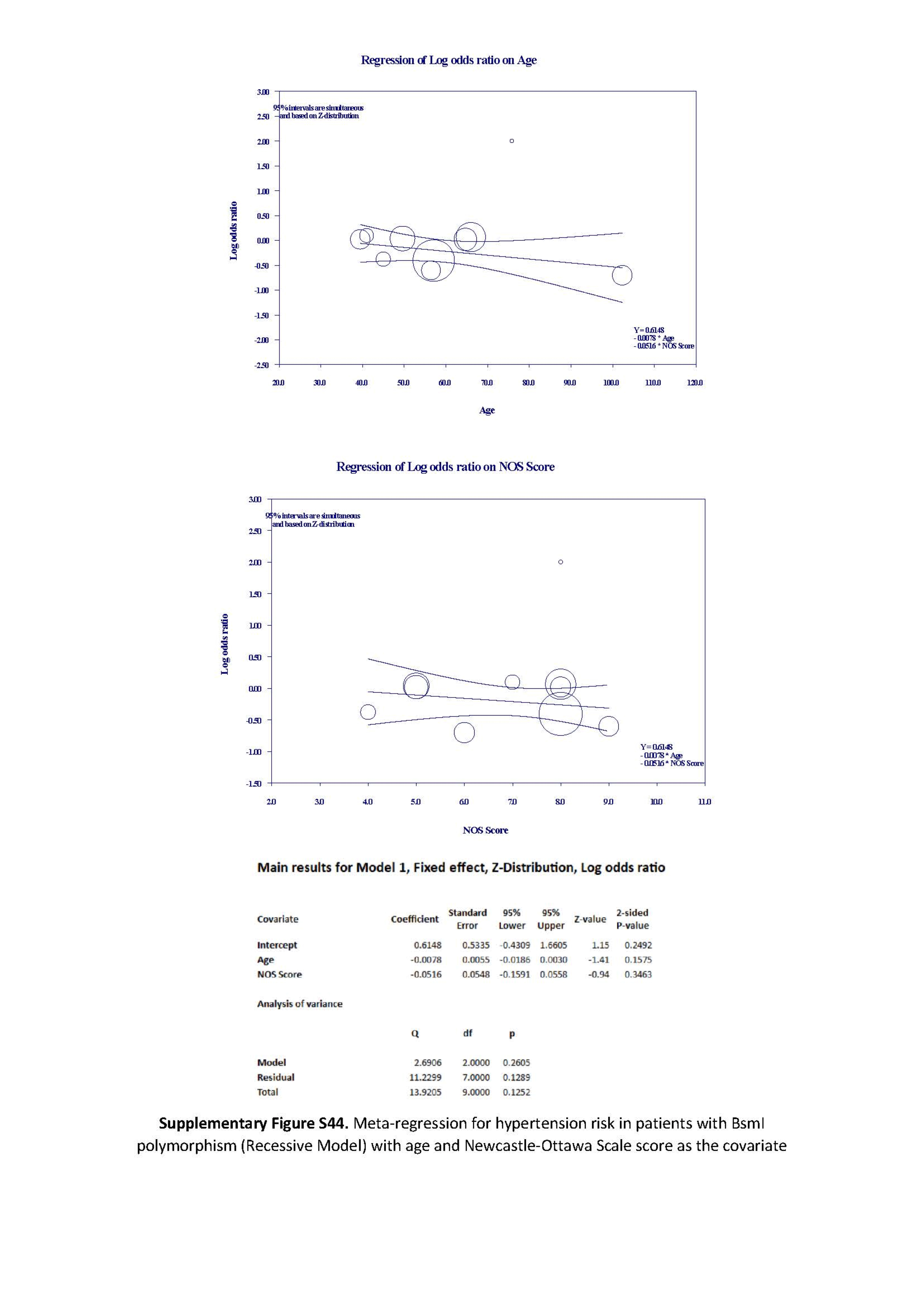

Supplement: S44 Fig — (JPG) [file pone.0314886.s048.jpg]

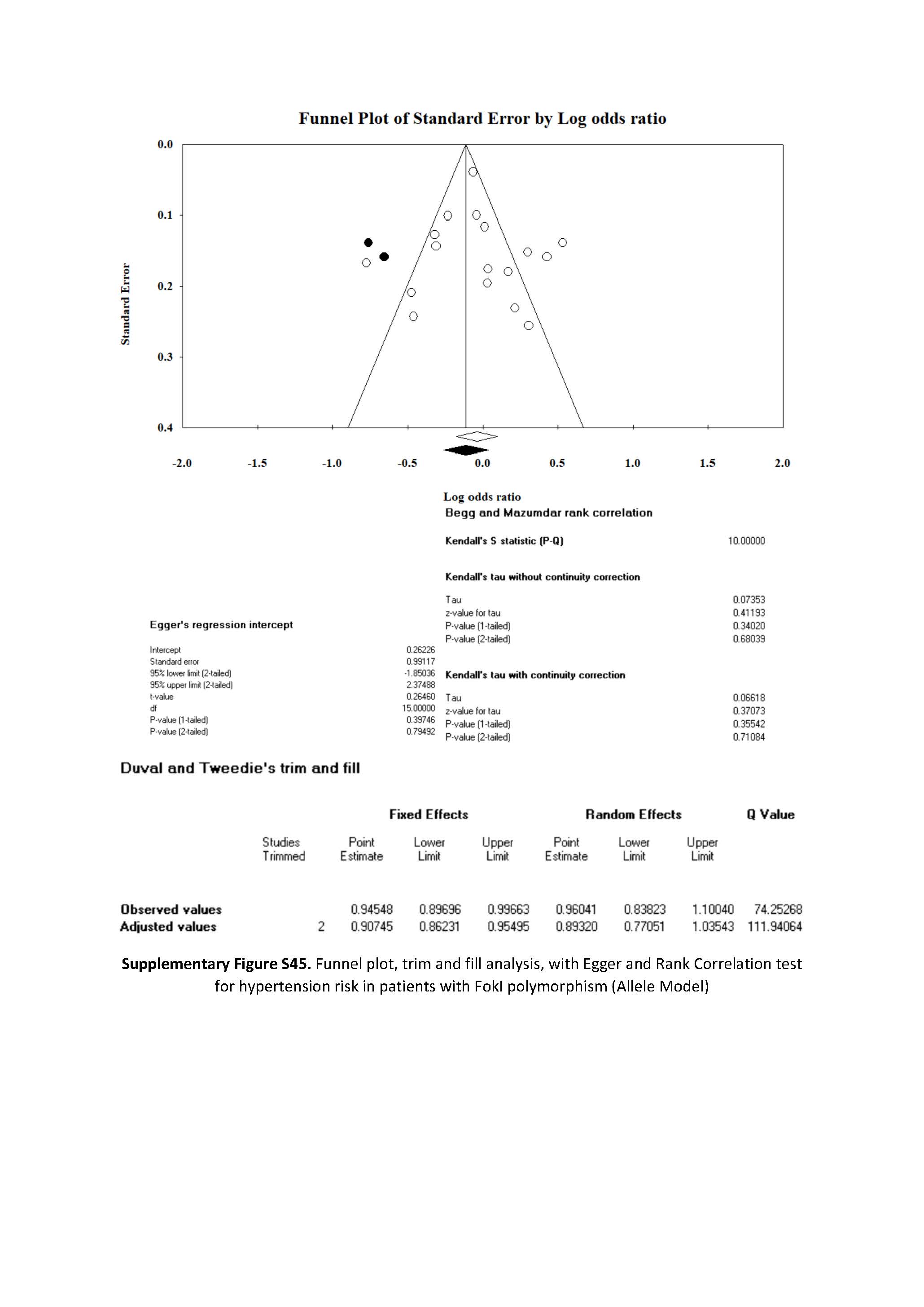

Supplement: S45 Fig — (JPG) [file pone.0314886.s049.jpg]

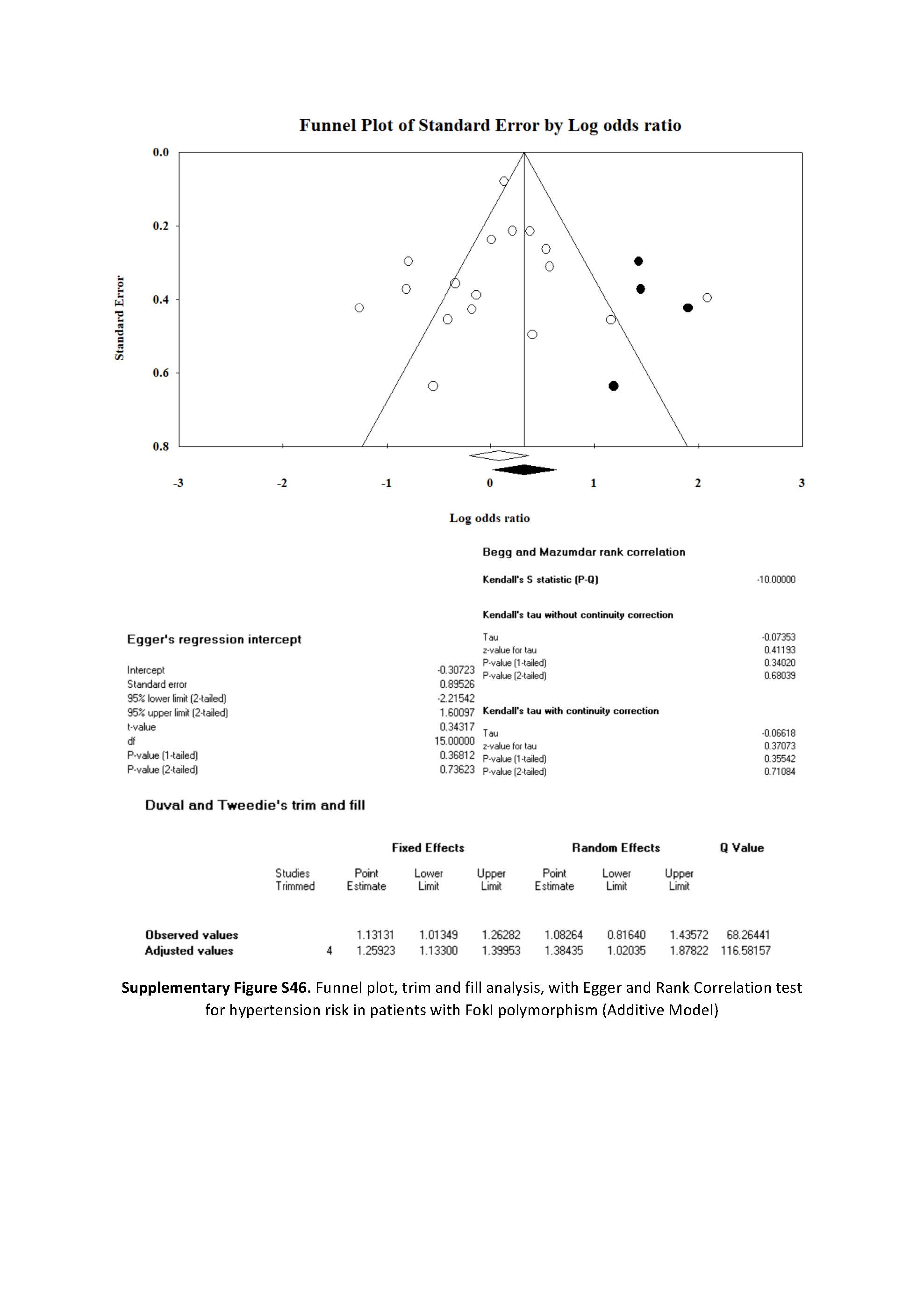

Supplement: S46 Fig — (JPG) [file pone.0314886.s050.jpg]

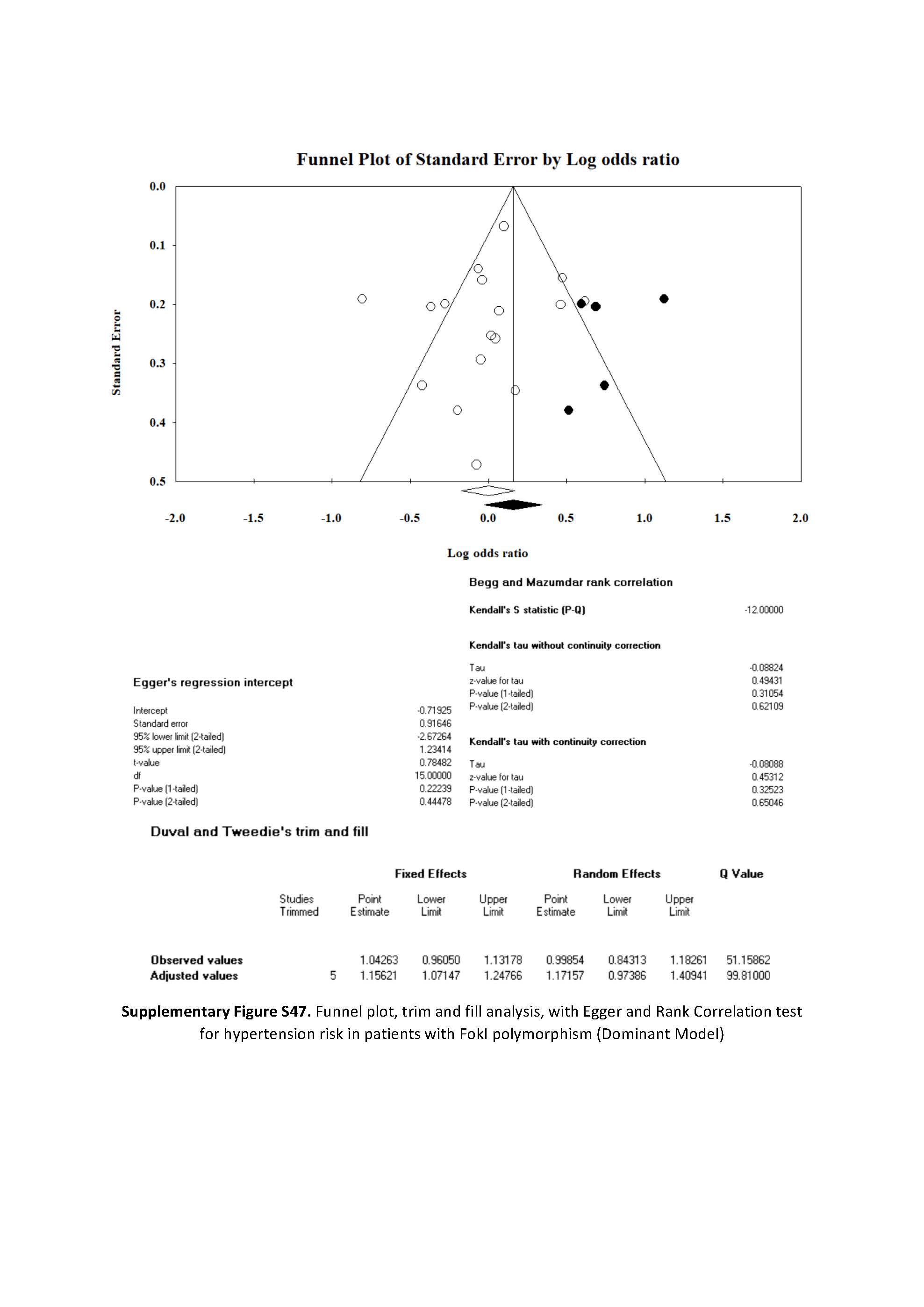

Supplement: S47 Fig — (JPG) [file pone.0314886.s051.jpg]

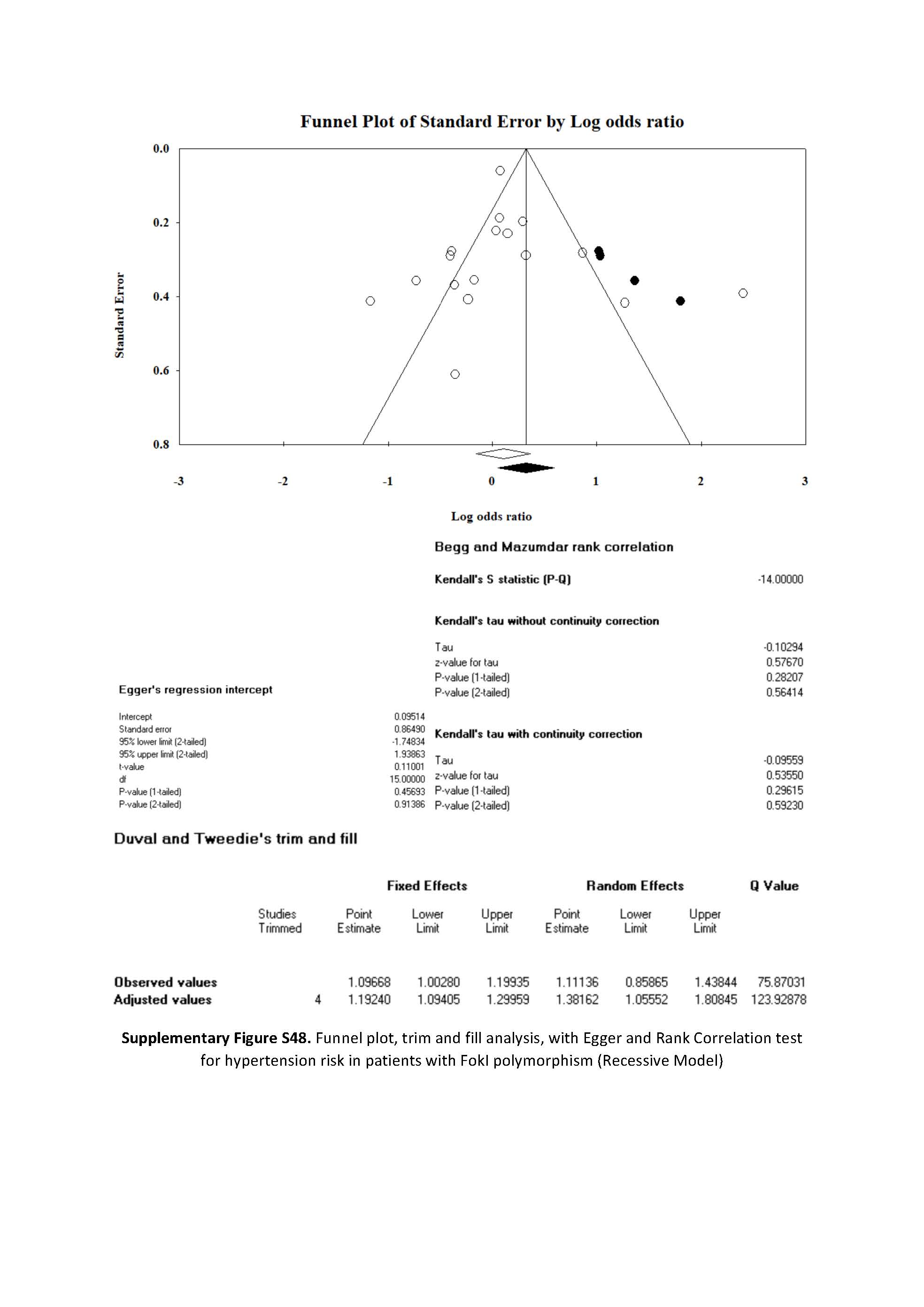

Supplement: S48 Fig — (JPG) [file pone.0314886.s052.jpg]

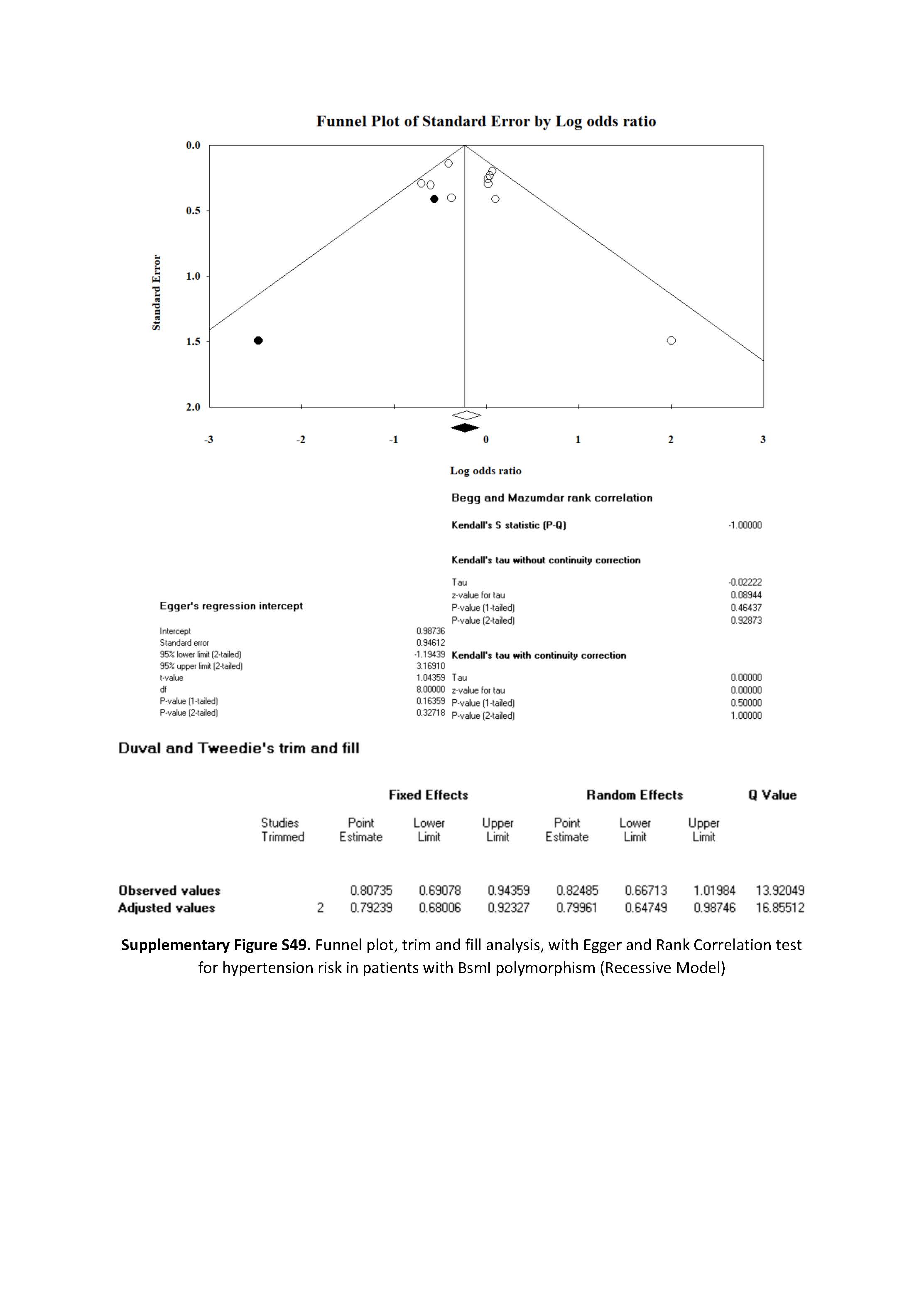

Supplement: S49 Fig — (JPG) [file pone.0314886.s053.jpg]
