# Supplementary material for: Association between vitamin D receptor gene polymorphism and essential hypertension: An updated systematic review, meta-analysis, and meta-regression
Source: PLoS One. 2024 Dec 23;19(12):e0314886. doi: 10.1371/journal.pone.0314886 (PMC11666036; doi:10.1371/journal.pone.0314886)
Supplement: Attachment — Submitted filename: RESPONSE TO EDITOR FOR SECOND EDITORIAL REVISION.docx [file pone.0314886.s054.docx]

**RESPONSE TO EDITOR (FOR SECOND EDITORIAL REVISION)**

1. "It appears that your ORCiD iD has not been validated in your Editorial Manager account and we are unable to proceed until that step is complete.

To validate your ORCiD iD in Editorial Manager, please follow the steps below:

a. In your Editorial Manager account, please go to ‘Update my Information’ (in the upper left-hand corner of the main menu), and click on the Fetch/Validate link next to the ORCiD field.

b. This link will take you to the ORCiD site and allow you to create a new iD or authenticate a pre-existing iD in Editorial Manager.

**AUTHORS RESPONSE: We have re-link the ORCID ID**

2. As required by our policy on Data Availability, please ensure your manuscript or supplementary information includes the following:

1. Thank you for including a table of studies identified in the literature search. We noted that there were only 23 studies included in this table however, your PRISMA flow chart suggests that 39 studies were assessed for eligibility. Please update this to be a numbered table of ALL studies identified in the literature search, including those that were excluded from the analyses and those that were included. Please also include headings for the table columns.

a. For every excluded study, the table should list the reason(s) for exclusion.

b. If any of the included studies are unpublished, include a link (URL) to the primary source or detailed information about how the content can be accessed.

**AUTHORS RESPONSE: We have revised this by attaching “Supplementary Files (Full Screening Results)”**

2. Thank you for providing a table of the RAW extracted data. Please update this table to include column headings and the following information for each study:

a. Name of data extractors and date of data extraction

b. Confirmation that the study was eligible to be included in the review.

c. All data extracted from each study for the reported systematic review and/or meta-analysis that would be needed to replicate your analyses.

**AUTHORS RESPONSE: We have revised this by re-attaching “Supplementary Files for RAW Data” and add new file “Supplementary Files RAW Data for Replication Analyses”**
